# ACSS2-TFEB axis acts as a critical regulator of the autophagic machinery in head and neck squamous cell carcinoma

**DOI:** 10.1038/s41419-025-07971-9

**Published:** 2025-08-26

**Authors:** Danhui Yin, Qian Yang, Shisheng Li, Yongchun Peng, Jianbo Zhang, Zuozhong Xie, Tengfei Fan

**Affiliations:** 1 Department of Otorhinolaryngology Head and Neck Surgery, The Second Xiangya Hospital, Central South University, 410011, Changsha, Hunan China; 2 Department of Oral and Maxillofacial Surgery, The Second Xiangya Hospital of Central South University, 410011, Changsha, Hunan China; 3 Department of Oral and Maxillofacial Surgery, Zhang Zhiyuan Academician Workstation, Hainan Western Central Hospital, Shanghai Ninth People’s Hospital, Danzhou, Hainan China

**Keywords:** Oral cancer, Oncogenes

## Abstract

Head and neck squamous cell carcinoma (HNSCC) has a high rate of metastasis and recurrence, and poses a considerable threat to patient survival. Autophagy, an intracellular degradation pathway, plays a crucial role in tumor progression; however, the underlying mechanisms of action remain unclear. This study aimed to explore the role of the ACSS2-TFEB axis in the regulation of autophagy and its impact on HNSCC cell proliferation, migration, invasion, and lysosomal function. HNSCC tumor tissues and cell lines were analyzed for ACSS2 protein expression. The effects of the ACSS2 knockdown on cell proliferation, migration, invasion, and autophagic flux were also assessed. The interaction between ACSS2 and transcription factor EB (TFEB) and its influence on lysosomal function were also examined. In this study, we found that ACSS2 protein expression was significantly upregulated and correlated with metastasis and poor prognosis. ACSS2 knockdown inhibited the proliferation, migration, and invasion of HNSCC cells, and disrupted autophagy flux, primarily by impairing lysosomal function. Additionally, ACSS2 was found to sustain autophagic flux through TFEB activation, a key regulator of the autophagy-lysosome pathway. TFEB activation promotes lysosomal function and autophagic flux, thereby facilitating tumor cell growth and metastasis. This study elucidated the molecular mechanism by which ACSS2 enhances HNSCC cell proliferation and invasion via TFEB activation. The ACSS2-TFEB axis is a potential therapeutic target for HNSCC and provides a foundation for the development of targeted therapies.

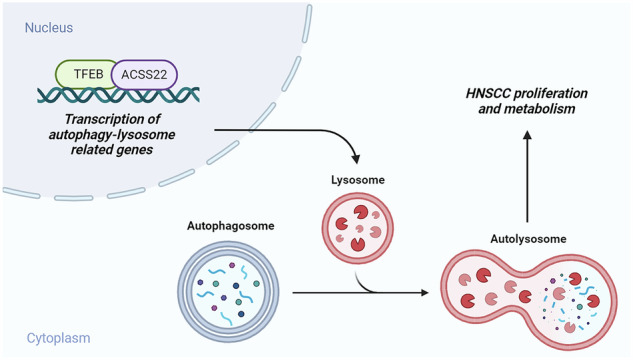

## Introduction

Head and neck squamous cell carcinoma (HNSCC), which originates in the oral cavity and pharyngeal and laryngeal mucosal epithelia, is the sixth most common type of cancer worldwide. Approximately 890,000 people are diagnosed with HNSCC annually, and its incidence is high in men [1]. The main risk factors for HNSCC include smoking, alcohol consumption, and human papillomavirus (HPV) infection [2]. The common symptoms include sore throat, hoarseness, dysphagia, and neck mass; however, the disease usually progresses to an advanced stage when the symptoms are notable [2]. HNSCC can be treated using various methods, including surgical resection, radiotherapy, chemotherapy, and immunotherapy [3]. Although recent progress has been made in comprehensive treatment, the prognosis of patients with advanced HNSCC remains poor, mainly because of the strong invasiveness, easy recurrence, and treatment resistance of tumors [4]. Therefore, an in-depth study of the pathogenesis of HNSCC and the search for new therapeutic targets is important to improve patient survival rates.

Autophagy is a degradation and recycling process in cells, which is crucial for maintaining the stability of the intracellular environment and promoting cell survival [5]. Under normal conditions, basal levels of autophagy are maintained in cells, and when cells are in states of metabolic abnormality or oxidative stress, they maintain their intracellular stability by digesting abnormal organelles or proteins [6]. Autophagy plays a dual role in tumor development. On the one hand, it removes damaged proteins and organelles, inhibiting tumor cell growth and proliferation; on the other hand, it can also provide energy and nutrients for the tumor cells, promoting their survival and metastasis [7]. Furthermore, autophagy exhibits a positive correlation with the emergence of drug resistance in HNSCC cells [8]. Numerous drugs associated with autophagy regulation have advanced to the stage of clinical application. Research findings indicate that the autophagic level in cancer cells is notably higher than that in normal tissues. Conventional radiotherapy and chemotherapy are capable of triggering protective autophagic responses in cancer cells. Consequently, directly targeting autophagy has emerged as a crucial strategy in cancer treatment. Representative drugs in this regard include chloroquine (CQ), bafilomycin A1 (Baf A1), and epigallocatechin gallate (EGCG). Notwithstanding, the therapeutic approach of suppressing autophagy may give rise to tumor resistance to radiotherapy or chemotherapy and facilitate tumor metastasis, thereby posing novel challenges to clinical treatment [9]. Therefore, an in-depth study on the mechanism of autophagy in HNSCC is critical for the identification of new therapeutic strategies.

Acyl-CoA synthetase short-chain family member 2(ACSS2) and Transcription factor EB (TFEB) were discovered in recent years; the two genes are associated with autophagy regulation. ACSS2 is a lipid metabolism enzyme that not only promotes the metabolism of tumor cells by converting long-chain fatty acids to the active form of acyl-CoA, but also participates in autophagy regulation. ACSS2 expression is abnormal and specific to a variety of tumors, and is closely related to tumor occurrence, development, and prognosis [10, 11]. ACSS2 expression in different tumors is opposite, which may be related to the complexity of autophagy [12]. One study reported that phosphorylated ACSS2 translocates to the nucleus and binds to and activates TFEB [13]. TFEB activation in the CLEAR, RRAGC, UVRAG, CSTB, M6PR, and IGF2R factor expression levels, strengthens lysosome gene expression, improves lysosome function and autophagy, and provides nutrition for tumor cells [12, 14]. Simultaneously, ACSS2 provides acetyl-CoA during histone H3 acetylation around lysosomes and autophagy gene promoters [14]. TFEB also directly binds to the promoter region of ACSS2 to regulate its expression. This interaction plays an important role in the occurrence and development of HNSCC and may be a potential target for treating HNSCC.

This study aimed to further explore the role of ACSS2 and TFEB in HNSCC and how they affect the biological behavior of tumor cells by regulating autophagy. We will conduct in vitro and in vivo experiments, verify the influence of ACSS2 and TFEB interaction on autophagy regulation, and further explore the underlying molecular mechanisms. Through an in-depth analysis of the mechanism of the ACSS2-TFEB axis in the regulation of autophagy in HNSCC, this study may provide a theoretical basis for the development of drugs targeting the ACSS2-TFEB axis and offer new strategies to improve the treatment effectiveness and survival rate of patients with HNSCC.

## Materials and methods

### Tissue samples

Surgical samples (including cancer tissues, paracancerous tissues, and lymph nodes) from 82 patients with HNSCC were collected at the Department of Otorhinolaryngology Head and Neck Surgery, the Second Xiangya Hospital, Central South University. The cases included in our study had not undergone any treatment prior to surgery. The collected tissue specimens were stored in liquid nitrogen until futher use. The specimens and clinical data were collected with the informed consent of the patients and their families, and were approved by the Ethics Committee of the Second Xiangya Hospital of Central South University (No. JBWKQA001).

### Immunohistochemistry(IHC)

Tumor tissues were harvested from nude mice and fixed in a 4% paraformaldehyde solution overnight. Subsequently, the tissues underwent paraffin embedding, sectioning, and routine slide preparation. The sections were then subjected to a series of procedures, including dewaxing, rehydration with gradient ethanol, antigen retrieval, treatment with a 3% hydrogen peroxide solution to block endogenous peroxidase activity, and thorough washing with PBS. Finally, nuclear staining of the sections was carried out using DAPI. Microscopic observation and image acquisition of the sections were performed using a digital pathology slide scanner (Pannoramic MIDI, 3DHISTECH, Hungary).

### Cell culture

Oral mucosal keratinocytes (OKC), human pharyngeal squamous cell carcinoma cell line (FaDu), human tongue squamous cell carcinoma cell lines (SCC9, SCC25, and CAL27), and human HNSCC cell line (SCC23) were used. Cells were cultured in DMEM/H (Gibco, C11995500BT) supplemented with 1% penicillin/streptomycin (Beyotime, C0222) and 10% fetal bovine serum (Viva Cell, C04001-50). Cells were maintained under standard culture conditions in a humidified incubator at 37 °C with 5% CO₂.

### Wound healing assay

Cells were seeded into 6-well plates at an appropriate density and cultured until they reached full confluence. Subsequently, a uniform mechanical wound model was established by making a linear scratch with a 200 μl pipette tip. Following this, the cell monolayers were gently washed three times with PBS to remove detached cells. The remaining adherent cells were then cultured in serum-free medium. At 0 h and 24 h after scratching, four randomly chosen fields of view within each well were photographed using an inverted microscope (magnification: 10×). Ultimately, image analysis software was employed to measure and calculate the changes in the distance between the wound edges.

### Cell clone formation assay

The cells were resuspended to 500 cells/mL of cell suspension concentration and 2 mL of this cell suspension was added to each well of 6-well plates. After culturing in an incubator for 14 d, the cells were removed, cleaned, fixed with poly-formaldehyde, stained with crystal violet (Solarbio, G1062), and photographed for analysis.

### Transwell cell invasion assay

Matrigel (Corning, 354234) was diluted with serum-free medium at a ratio of 1:5, and 50 μL was spread on the upper chamber of the Transwell chamber (Corning, 3422). After the gel solidified, the transfected cells were resuspended in serum-free medium, and 200 μL of cell suspension (approximately 5 × 10^4^ cells) was added to the upper chamber of the Transwell. After 48 h of culturing, the cells were fixed with 95% ethanol and stained with 0.5% crystal violet (Solarbio, G1062). The number of invasive cells were observed under a microscope. Four fields were randomly selected and the average number of cells were calculated.

### shRNA transfection

shRNA primers targeting ACSS2 and ATG5 were synthesized by Ribobio (Guangzhou, China) for gene knockdown. The shRNA sequence was embedded into the vector using T4 ligase. For lentivirus packaging, 293T cells were seeded at a density of 40–50% and subsequently co-transfected with an shRNA-containing lentiviral vector, envelope plasmid, and packaging plasmid. After transfection, the virus-rich supernatants were collected and used to infect SCC9 and CAL27 cells. Following trypsin-mediated generation of single-cell suspensions, cells were divided into the negative control group (NC) and experimental knockdown groups. These groups were transduced with either negative control lentivirus or lentiviral shRNA vectors targeting ACSS2/ATG5, respectively. After transfection, subsequent antibiotic selection and clonal purification procedures yielded stable ACSS2/ATG5 double-knockout cell lines. β-catenin expression was verified using real-time polymerase chain reaction (PCR) and western blot.

### Plasmid transfection

SCC9/CAL27 cells were seeded at a density of 2 × 10^5^ cells/well in 6-well plates. Lipofectamine 3000 transfection reagent (Invitrogen, L3000015) and the TFEB overexpression plasmid were diluted in serum-free medium. Lipofectamine 3000 transfection reagent (250 μL/well) and plasmids (2 μg/well) were added to 6-well plates, and the corresponding plasmid was added to each group. Six hours after transfection, the medium was changed for 48 h, and the transfection efficiency was observed under a fluorescence microscope.

### Immunofluorescence assay

Cells from the shACSS2 and NC groups in the logarithmic growth phase were seeded onto sterile cover slides in 6-well plates and cultured in an incubator. After 48 h, the medium, PBS washed three times with PBS. The plates were fixed in 4% paraformaldehyde (Beyotime, P0099) for 15 min and washed thrice with PBS. Membranes were lysed with 0.2% Triton X-100 (Sigma-Aldrich, T8787) (prepared in PBS) for 15 min at room temperature and washed three times with PBS. After blocking with 5% goat serum for 1 h at room temperature, the blocking solution was discarded, and primary antibodies were added and incubated overnight at 4 °C. The cells were then washed three times with PBS. Fluorescent secondary antibodies were added and incubated at room temperature in the dark for 1 h followed by washing with PBS for three times. DAPI (Beyotime, C1005) was added, and the cells were incubated in the dark for 3 min. After washing with PBS, slides were sealed, observed under a fluorescence microscope, and photographed.

### Real-time quantitative PCR

Total RNA was extracted from the tumor tissue using the RNAisoPlus (TaKaRa, 9109), and its purity and concentration were determined. cDNA was synthesized by reverse transcription using PrimeScript RT Reagent Kit (TaKaRa, RR047A) and detected by RT-PCR. The reaction conditions were 95 °C for 1 min, 95 °C for 20 s denaturation, 58 °C for 20 s annealing, and 72 °C for 20 s extension for 45 cycles. The relative mRNA expression level of ACSS2 was calculated by the 2^-ΔΔCt^ method with GAPDH as the internal control.

### Western blot

Cellular lysates were prepared using RIPA lysis buffer (Beyotime, P0013B) across all experimental groups. Following complete lysis, samples were centrifuged at 20,000 g for 20 min at 4 °C. The resultant supernatants were maintained on ice prior to protein quantification via BCA (Beyotime, P0011) assay. Quantification was followed by protein electrophoresis protein transfer, blocking, antibody incubation, and ECL luminescence visualization using the Chemi Doc XRS^+^system. ImageJ software was used for the gray-level analysis.

### RFP-GFP-LC3 double-labeled adenovirus assay

RFP-GFP-LC3 double-labeled adenovirus (Invitrogen, P36239) was added to shASCC2 cells in the logarithmic growth phase and to the ASCC2 cell culture medium at the recommended multiplicity of infection (MOI) values. The RFP-GFP-LC3B kit is a tool extensively utilized in autophagy research. Under acidic conditions, the green fluorescence signal of GFP undergoes quenching, whereas the red fluorescence signal of RFP remains constant. Upon the fusion of autophagosomes and lysosomes, the green fluorescence attenuates markedly, and the red fluorescence emerges as the primary signal. Following treatment with the RFP-GFP-LC3 kit, the cells were placed in an incubator for culturing. After 24 to 48 h of infection, cells were visualized using fluorescence microscopy for autophagosome formation (yellow dots, GFP, and RFP co-localization) and autophagosome fusion with lysosomes (red dots, GFP quenched with only the RFP signal remaining). The acquired fluorescence images were analyzed using ImageJ software, and the numbers of yellow and red dots were counted to quantify autophagosome formation and degradation.

### Xenograft mouse experiments

Male Balb/c inbred mice of SPF grade, 6–8 weeks old, approximately 20 g, were routinely housed in cages with 5 mice each and the animals were fed with access to water freely during the experiment. Mice were randomly allocated into six groups. Specifically, mice in Group 1 and Group 2 were subcutaneously injected with CAL27 cells transfected with shACSS2 or a negative control (NC). Mice in Group 3 and Group 4 were subcutaneously injected with SCC9 cells transfected with shACSS2 or NC. Mice in Groups 5 and 6 were subcutaneously injected with CAL27 cells transfected with NC. Subsequently, mice in Groups 1 to 4 were fed a standard diet. Mice in Group 5 were administered 25 mg/kg of the ACSS2 inhibitor VY - 3 - 135 (Selleck, Cat. No. S8588) via oral gavage daily. As a control, mice in Group 6 were administered the same volume of normal saline via oral gavage. Following standardized aseptic preparation of the right axillary region in all groups, a precisely measured 0.1 mL aliquot of group-specific cell suspension (5×10^7 cells/mL) was administered via subcutaneous injection to each mouse under specific pathogen-free conditions. The tumor volume was measured every three days, and the mice were sacrificed day 27. Tumors were isolated, measured, photographed, and subsequently subjected to IHC or WB experiments to evaluate the expression of related protein.

### Statistical analysis

SPSS22.0 software was used for statistical analysis. Kaplan-Meier survival analysis was used to study the relationship between ACSS2 expression levels and the overall survival of patients. An independent samples t test was used for comparison between two groups, and a one-way analysis of variance (ANOVA) was used for comparison between multiple groups. *p* < 0.05 was considered statistically significant.

## Results

### Relationship between ACSS2 expression level and survival time of patients with HNSCC

**Fig. 1:**
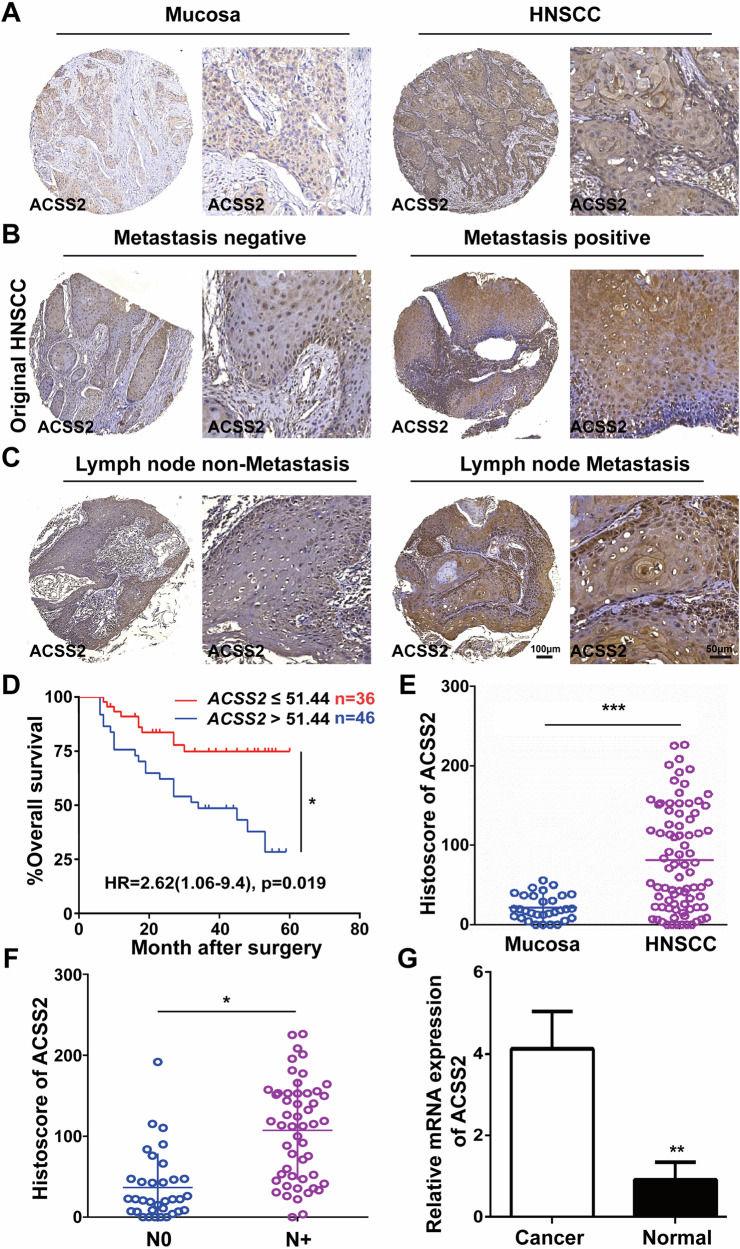
ACSS2 upregulation in HNSCC tissues and cells. **A** Representative IHC images of ACSS2 in normal oral mucosal tissues and HNSCC. Scale bar=100 μm. **B** Representative IHC images of ACSS2 in metastatic positive and metastatic negative OSCC tissues. Scale bar = 100 μm. **C** Representative IHC images of ACSS2 in lymph node tissue of patients with HNSCC with positive lymph node metastasis. Scale bar = 100 μm. **D** Kaplan–Meier survival in patients with high and low ACSS2 expression of HNSCC, with a cutoff of the median ACSS2 value. **E** Histoscore of ACSS2 in normal oral mucosal tissue and HNSCC. **F** Histoscore of ACSS2 in lymph node tissue of patients with HNSCC with positive and negative lymph node metastases. **G** The expression of ACSS2 in patients with HNSCC and normal tissues was determined by qPCR. *p < 0.05, **p < 0.01, ***p < 0.001.

By comparing the clinicopathological characteristics of patients with HNSCC with the ACSS2 expression levels, it can be known that patients with high ACSS2 expression presented with later tumor stages and extended lymph node metastases (Table 1). To explore the significance in HNSCC, IHC, and ACSS2 histoscore assessment in patients with HNSCC carcinoma tissue and the expression level of normal oral mucosa tissues. The results showed that compared with normal oral mucosa tissues, ACSS2 expression in HNSCC tissues was significantly increased (*p* < 0.001, Fig. 1A, E) and ACSS2 expression was positively correlated with TNM stage and lymph node metastases (Fig. 1B, C, F). Similarly, RT-qPCR results showed that the mRNA expression level of ACSS2 was significantly increased in OSCC tissues (Fig. 1G). Kaplan–Meier survival analysis showed that the ACSS2 high expression of the overall survival rate was significantly lower than the low expression group (*p* = 0.019, Fig. 1D).

**Table 1:** Relationship with the patient’s clinicopathological characteristics and ACSS2 expression alteration in HNSCC patients.

Variables	No.	ACSS2 expression		*p* value
		High	Low	
Gender				0.2586
Male	57	22	35	
Female	25	13	12	
Age, years				0.608
<60	54	34	20	
≥60	28	16	12	
Smoking				0.926
Yes	55	23	32	
No	27	11	16	
Drinking				0.825
Yes	39	20	19	
No	43	21	22	
Tumor stage				**0.025**
3–4	41	19	22	
1–2	41	29	12	
Clinical stage				**0.000**
3–4	36	7	29	
1–2	46	27	19	
Lymph node metastasis				**0.012**
+	35	9	26	
—	47	25	22	

Bold values identify statistical significance.

### Knockdown of ACSS2 inhibited proliferation, migration and invasion of HNSCC cells

**Fig. 2:**
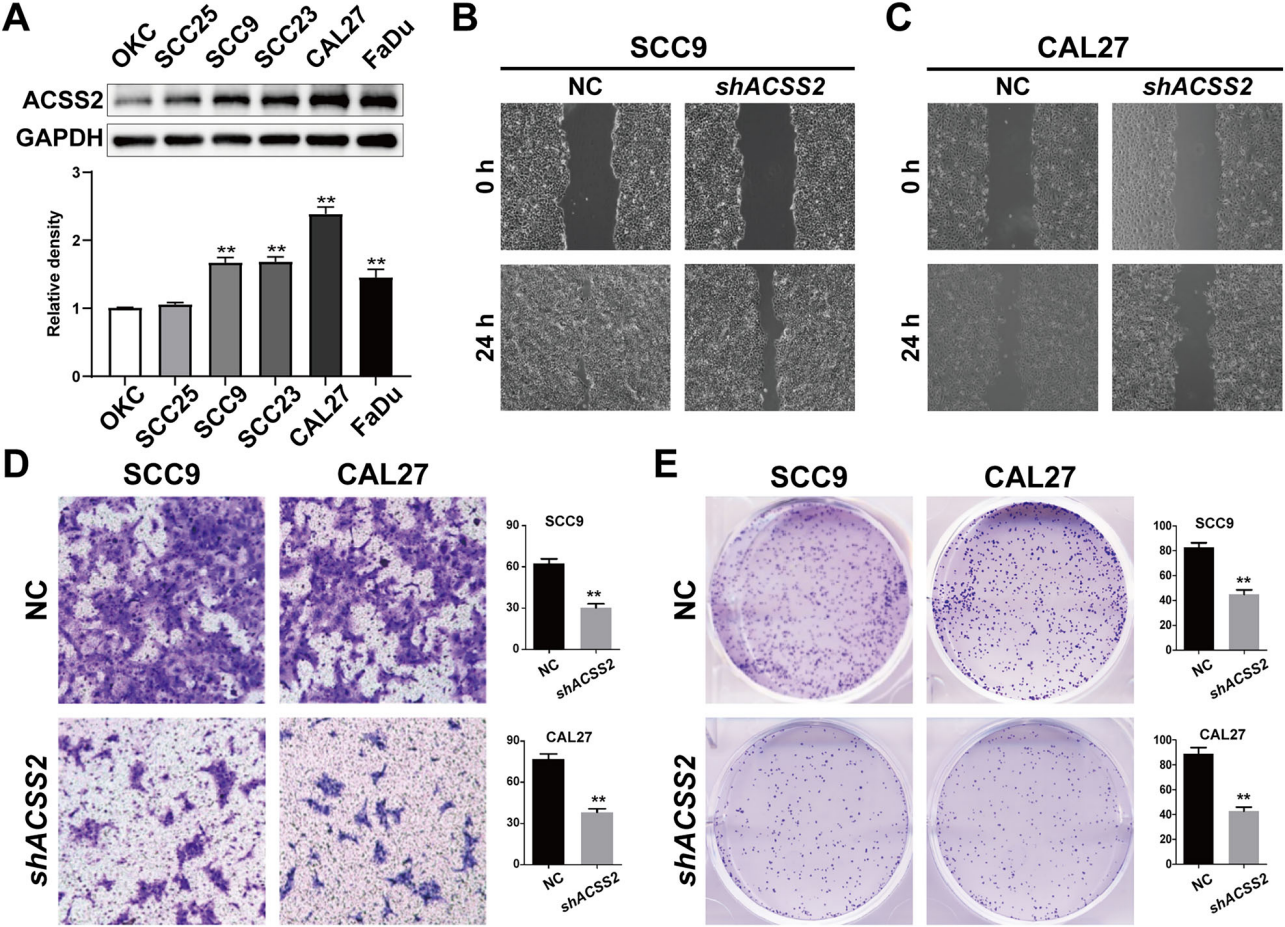
Effects of ACSS2 knockdown on proliferation, migration, and apoptosis of HNSCC cells. **A** Protein expression levels of ACSS2 in OKC, SCC25, SCC9, SCC23, CAL27, and FaDu and quantitative statistical results. **B** Wound healing results of ACSS2 knockdown/NC SCC9 at 0 h and 24 h. Scale bar = 100 μm. **C** Wound healing results of ACSS2 knocked down/control CAL27 at 0 h and 24 h. Scale bar = 100 μm. **D** Transwell invasion images of ACSS2 knocked down/NC SCC9 and CAL27 at 72 h and quantified statistical results. Scale bar = 100 μm. **E** ACSS2 knockdown/NC SCC9 and CAL27 clone formation images and quantified statistical results. **p < 0.01.

Western blotting was used to detect the protein expression levels of ACSS2 in OKC, SCC25, SCC9, SCC23, CAL27, and FaDu cells. The results showed that the expression levels of ACSS2 in SCC9, SCC23, CAL27, and FaDu cells were significantly higher than those in OKC (Fig. 2A). The cell lines with the most notable differences, SCC9 and CAL27, were selected for subsequent experiments. To comprehensively investigate the impact of the ACSS2 gene on the proliferation, invasion, and migratory capabilities of head and neck squamous cell carcinoma (HNSCC) cells, in this study, we established stable shACSS2-transfected knockdown cell lines along with their corresponding NC cell lines. shACSS2 SCC9 cell to experiment group, and NC group, within 24 h of scratch width measurement, calculation of cell migration rate, groups showed that the shACSS2 group had significantly decreased cell migration ability, and the difference was statistically significant (Fig. 2B). Transwell experimental detection cell invasion ability. After 72 hours of the experiment, the cells were stained and the number of cells that migrated to the lower chamber was counted. Results showed shACSS2 group cell invasion ability significantly reduced, the difference was statistically significant (Fig. 2D). Furthermore, the findings of the clonogenic assay demonstrated that the number of SCC9 cell colonies in the shACSS2 group was significantly lower than that in the NC group, and the difference was statistically significant (Fig. 2E). This phenomenon was also observed in CAL27 cells (Fig. 2C, D, E). Parallel experiments were conducted in the SCC25 cell line, which exhibits low endogenous ACSS2 expression levels. Following ACSS2 overexpression, we observed phenotypic effects consistent with those demonstrated in both SCC9 and CAL27 cell lines (Fig. S1A–E).

### Knockdown of ACSS2 inhibited autophagy flux in HNSCC cells

**Fig. 3:**
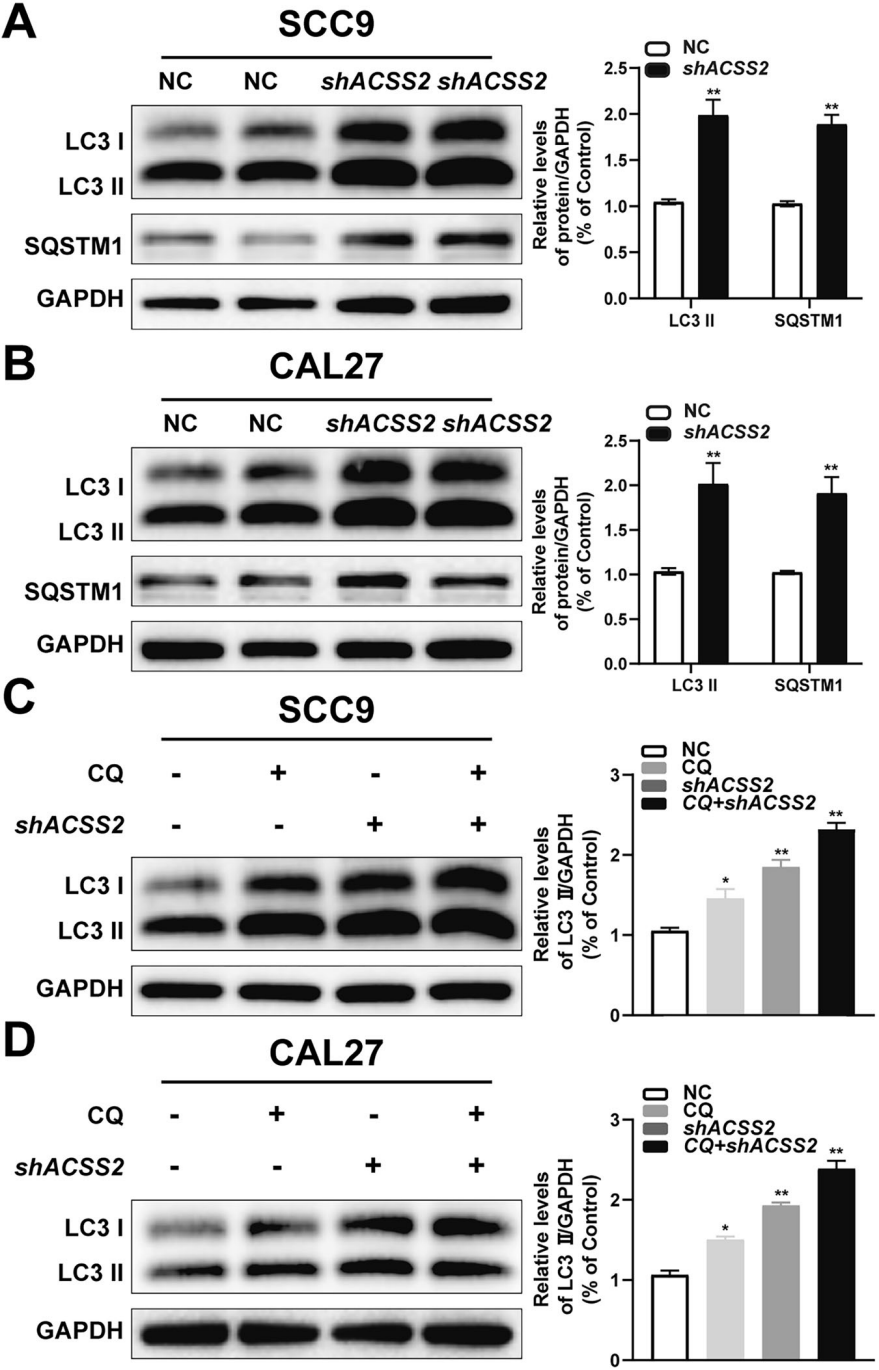
Inhibition of autophagy flux and lysosome function in HNSCC cells by ACSS2 knockdown. **A** The expression levels of autophagy marker proteins LC3I, LC3II, and p62 in SCC9 cells transfected with sh-ACSS2 were measured by Western blotting and quantified statistical results. **B** The expression levels of autophagy marker proteins LC3I, LC3II, and p62 in CAL27 cells transfected with sh-ACSS2 were measured by Western blotting and quantified statistical results were obtained. **C** The expression levels of LC3I and LC3II in SCC9 cells transfected with sh-ACSS2 after chloroquine treatment were measured by western blot and the quantitative statistical results were obtained. **D** The expression levels of LC3I and LC3II in CAL27 cells transfected with sh-ACSS2 after the addition of chloroquine were measured by western blot and the quantitative statistical results were obtained. *p < 0.05, **p < 0.01.

Considering the critical role of autophagy in tumor cell function, we further investigated the effect of ACSS2 KD on autophagic flux in HNSCC cells. LC3 plays a crucial role in autophagosome formation. During this process, cytoplasmic LC3 (LC3-I) undergoes proteolytic cleavage of its C-terminal peptide, followed by conjugation with phosphatidyl-ethanolamine to form lipidated LC3 (LC3-II), which is specifically recruited to autophagosomal membranes. The LC3-II protein expression serves as a biochemical marker of autophagosome biogenesis. Meanwhile, p62/SQSTM1, a selective autophagy substrate, is progressively degraded through autophagic-lysosomal pathways. Thus, decreased p62 protein levels directly correlate with enhanced autophagic flux. When the level of LC3II increases while that of P62 decreases, it indicates that the autophagic flux remains unperturbed. If both the levels of LC3II and P62 increase, it suggests that the initiation of autophagy is normal. This phenomenon may be attributed to the inhibition of autophagosome degradation, thereby impeding the autophagic flux. Western blotting results showed that the LC3II and P62 expression were significantly upregulated in both SCC9 and CAL27 cells after ACSS2 knockdown (Fig. 3A, B). Chloroquine (CQ), an autophagic lysosome inhibitor, can inhibit lysosomal activity, and the addition of CQ to some extent exacerbated the increase in the LC3II expression induced by shACSS2 (Fig. 3C, D), indicating that ACSS2 knockout inhibited autophagy flux in HNSCC cells.

### ACSS2 KD does not inhibit autophagosome formation or maturation in HNSCC cells

**Fig. 4:**
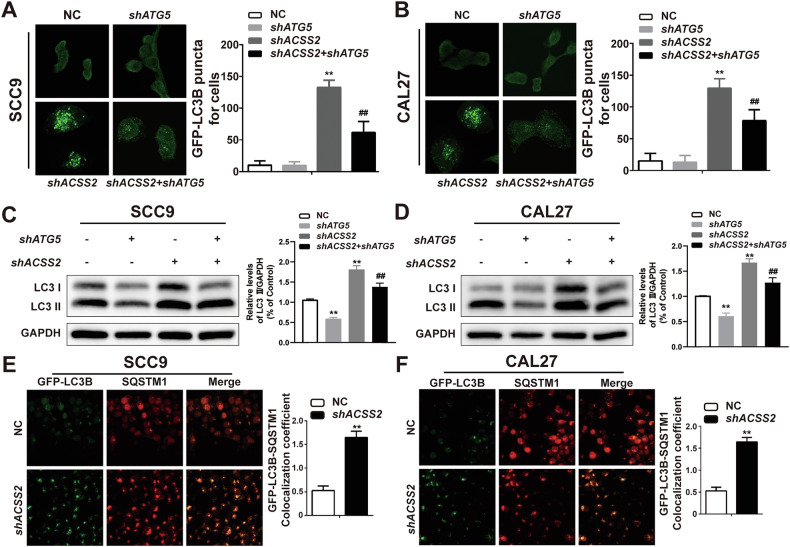
ACSS2 KD does not inhibit autophagosome formation or maturation in HNSCC cells. **A** GFP-LC3B immunofluorescence images and fluorescence intensity in SCC9 cells transfected with ACSS2 KD and/or sh-ATG5. Scale bar = 5 μm. **B** GFP-LC3B immunofluorescence images and fluorescence intensity in CAL27 cells transfected with ACSS2 KD and/or sh-ATG5. Scale bar = 5 μm. **C** The expression levels of LC3I and LC3II in SCC9 cells transfected with ACSS2 KD and/or sh-ATG5 were measured by western blotting and quantified statistical results. **D** The expression levels of LC3I and LC3II in CAL27 cells transfected with ACSS2 KD and/or sh-ATG5 were measured by western blotting and quantified statistical results. **E** Immunofluorescence co-localization images and quantitative statistical results of GFP-LC3B and SQSTM1 in SCC9 cells of ACSS2 KD. Scale bar = 20 μm. **F** Immunofluorescence co-localization images of GFP-LC3B and SQSTM1 in SCC9 cells of ACSS2 KD and quantitative statistical results. Scale bar = 20 μm. **p < 0.01, ^##^*p* < 0.01.

Autophagy-related gene 5 (ATG5) plays a central role in the formation of the autophagy body. During the initiation of autophagy, the ATG5-ATG12-ATG16L complex localizes to the assembly site of the autophagosome to facilitate the expansion and maturation of the membrane structure and finally forms a complete autophagosome. We transfected ATG5 shRNA plasmids into SCC9 and CAL27 cells, with or without ACSS2 knockdown. The results demonstrated that subsequent to an additional knockdown of ATG5, the fluorescence intensity of GFP-LC3B in HNSCC cells with ACSS2 knockdown was markedly diminished, and the LC3II declined. This outcome implies that shATG5 interference can effectively suppress the phenomenon of LC3II accumulation induced by ACSS2 knockdown. Moreover, this result suggests that the knockdown of ACSS2 has no impact on the formation of autophagosomes within HNSCC cells (Fig. 4A–D). SQSTM1 functions as a cargo receptor during autophagy and is a key component of mature autophagosomes. It binds to ubiquitinated substrates and targets them to autophagosomes for degradation. SQSTM1 also contains an LC3 interaction area (LIR) that allows it to directly combine with the LC3 protein, thereby bringing the ubiquitin substrate for autophagy in the body. We conducted a quantitative assessment of the maturation of autophagosomes via the localization assays of GFP-LC3B and SQSTM1. The results revealed that, in comparison with the control group, the localization coefficient in the ACSS2 knockdown group was markedly elevated, and it exhibited a positive correlation with the increase in the number of autophagosomes. This finding suggests that the knockdown of ACSS2 does not exert a significant impact on the process of mature autophagy in HNSCC cells (Fig. 4E, F).

### ACSS2 KD does not interfere with autophagosome-lysosome fusion in HNSCC cells

**Fig. 5:**
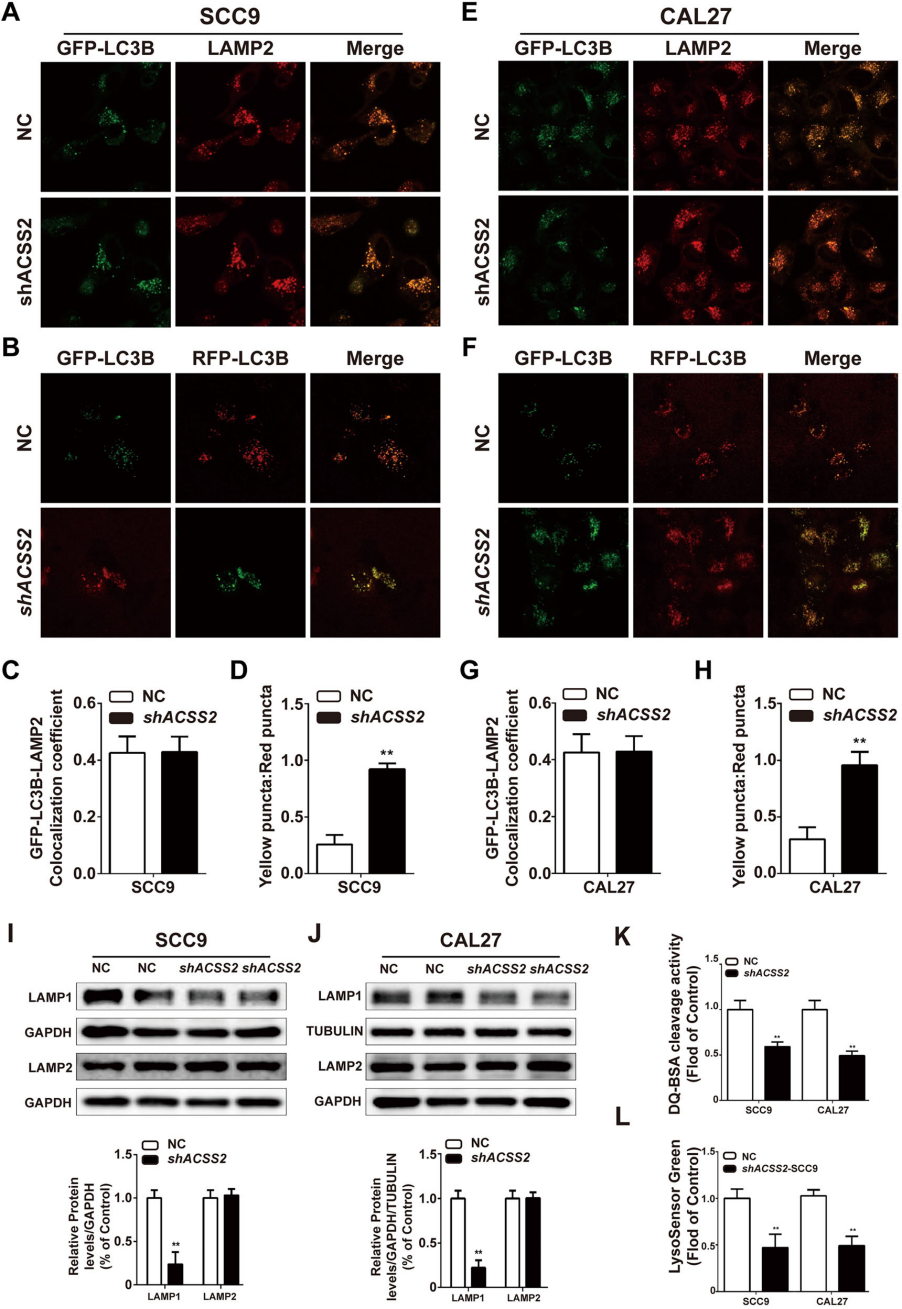
ACSS2 KD did not interfere with autophagosome lysosome fusion in HNSCC cells. **A** Immunofluorescence co-localization images of GFP-LC3B and LAMP2 in SCC9 cells of ACSS2 KD and (**C**) quantitative statistical results. Scale bar = 10 μm. **B** Immunofluorescence co-localization images of GFP-LC3B and LAMP2 in SCC9 cells of ACSS2 KD and (**D**) quantitative statistical results. Scale bar = 10 μm. **E** Immunofluorescence co-localization images of GFP-LC3B and LAMP2 in CAL27 cells of ACSS2 KD and (**G**) quantitative statistical results. Scale bar = 10 μm. **F** Immunofluorescence co-localization images of GFP-LC3B and LAMP2 in CAL27 cells of ACSS2 KD and (**H**) quantitative statistical results. Scale bar = 10 μm. **I** The expression levels of lysosomal membrane marker proteins LAMP1 and LAMP2 in SCC9 cells transfected with sh-ACSS2 were measured by Western blotting. **J** The expression levels of lysosomal membrane marker proteins LAMP1 and LAMP2 in CAL27 cells transfected with sh-ACSS2 were measured by Western blotting and the quantitative statistical results were obtained. **K** Fluorescence intensity of DQ-BSA staining in shACSS2 or unknocked SCC9 and CAL27 cells. **L** Fluorescence intensity of LysoSensor Green in shACSS2 or control SCC9 and CAL27 cells. **p < 0.01.

In the late stages of autophagy, autophagy fusion with lysosomes forms autophagy-lysosomes, and degrades the content of autophagy in the body. LC3II and LAMP2 are the hallmark membrane proteins of autophagosomes and lysosomes, respectively. When autophagolysosomes are formed, LC3II and LAMP2 show intracellular colocalization. There was no significant change in the colocalization ratio of LC3II and LAMP2 between SCC9 and CAL27 cells after ACSS2 knockdown compared to that in the NC group (Fig. 5A, C, E, G). RFP-GFP-LC3 double standard adenoviruses can be used to detect the formation of autophagosomes (yellow point, namely GFP and RFP), and the autophagosomes can be integrated with the lysosome (red point, namely GFP only after quenching RFP signals). In an acidic environment, green fluorescent protein (GFP) degrades, and at this time, autolysosomes appear as red spots. If autophagosomes and lysosomes could fuse normally, the red fluorescence would be more intense than the yellow fluorescence. If the downstream pathway of autophagy was blocked and the fusion of autophagosomes and lysosomes was abnormal, yellow fluorescence was observed. The results showed that shACSS2 after two puncta cells in the yellow and red puncta ratio significantly increased compared to the NC group (Fig. 5B, D, F, H).

### Knockdown of ACSS2 inhibits lysosomal function of HNSCC cells

LAMP1 and LAMP2 are in the lysosome membrane on the surface of the lysosomal markers. SCC9 and CAL27 cells after knockdown of ACSS2; LAMP1 protein expression level decreased significantly, but did not significantly affect LAMP2 (Fig. 5I, J). DQ™ Red BSA is a bovine serum albumin conjugated with a red fluorescent dye. When DQ™ Red BSA is taken up by lysosomes and degraded, its red fluorescence is enhanced. Compared with the control group, ACSS2 KD HNSCC cells in DQ ™ Red fluorescence intensity of BSA decreased (Fig. 5K). LysoSensor Green DND-189 is a green fluorescent probe used to measure the pH of acidic organelles such as lysosomes. The fluorescence of the dye was enhanced in acidic environments, and its fluorescence signal showed a pH-dependent enhancement. This probe exhibits accumulation within acidic organelles. Subsequent to the knockdown of ACSS2, a notable reduction in the fluorescence intensity of LysoSensor Green was observed in both SCC9 and CAL27 cells. These results suggest that knockdown of ACSS2 inhibits lysosomal function in HNSCC cells (Fig. 5L). To investigate the functional interplay between ACSS2 and autophagy in tumor cell behavior, we systematically modulated autophagic activity in shACSS2 cells using pharmacological agents: chloroquine as an autophagy inhibitor and Torin 1 as an autophagy inducer. Through comprehensive functional characterization, including scratch wound healing assays, Transwell invasion assays, and clonogenic survival assays, we observed that autophagy inhibition with CQ exacerbated the phenotypic consequences of ACSS2 depletion, resulting in significant suppression of both proliferative capacity and migratory potential. Conversely, Torin 1-mediated autophagy activation partially rescued the impaired cellular functions in shACSS2 cells (Fig. S2A–E).

### TFEB is a key factor in the regulation of lysosomal function mediated by ACSS2 in the progression of HNSCC

To investigate the mechanism by which ACSS2 interferes with lysosomal function in HNSCC cells, we proteomically compared the differential proteins of SCC9 and Cal27 cells with and without ACSS2 knockout, and found that shACSS2 led to a significant decrease in TFEB mRNA level(Fig. S3A, B). Western blotting results further demonstrated decreased expression levels of TFEB proteins in the shACSS2 group (Fig. S3C, D). This suggests that TFEB is closely associated with ACSS2-induced lysosomal function changes.

**Fig. 6:**
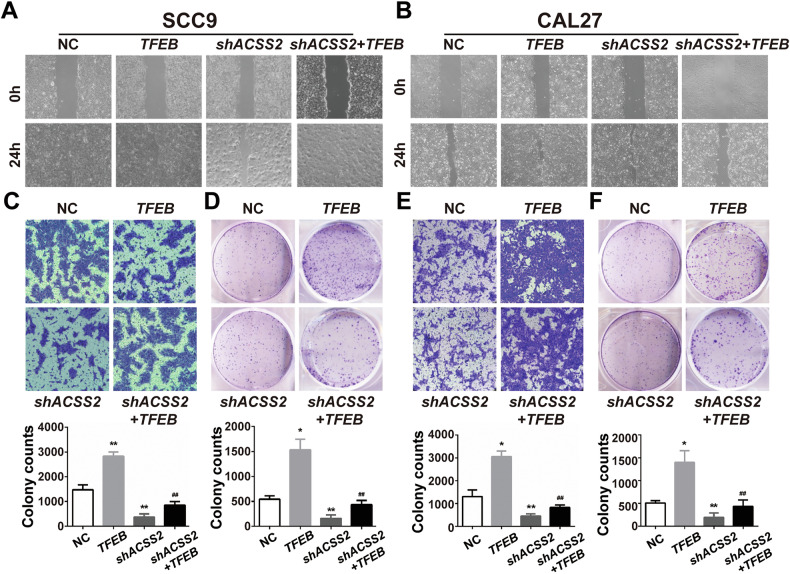
Effects of TFEB overexpression on proliferation, migration, and apoptosis of ACSS2-knockdown HNSCC cells. **A** Wound healing results of TFEB overexpression and/or ACSS2-knockdown SCC9 at 0 h and 24 h. Scale bar = 100 μm. **B** Wound healing results of TFEB overexpression and/or ACSS2 knockdown of CAL27 at 0 h and 24 h. Scale bar=100 μm. **C** TFEB overexpression and/or ACSS2 knockdown of SCC9 at 72 h Transwell invasion images and quantified statistical results. Scale bar = 100 μm. **D** TFEB overexpression and/or ACSS2 knockdown of CAL27 at 72 h Transwell invasion images and quantified statistical results. **E** Cloning of SCC9 with TFEB overexpression and/or ACSS2 knockdown generates images and quantified statistical results. Scale bar = 100 μm. **F** Cloning of CAL27 with TFEB overexpression and/or ACSS2 knockdown generates images and quantified statistical results. *p < 0.05, **p < 0.01, ^##^*p* < 0.01.

**Fig. 7:**
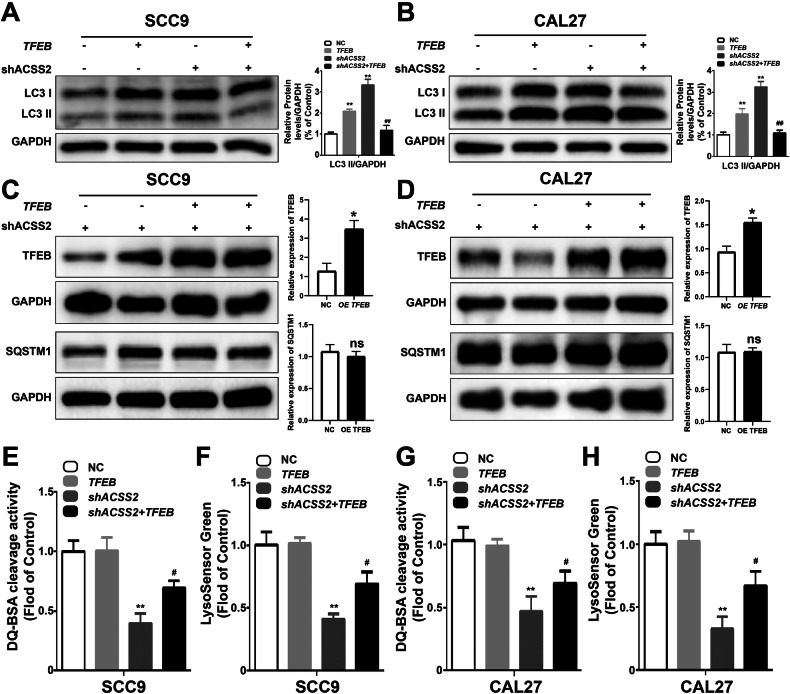
Effects of TFEB overexpression on autophagy flux and lysosome function of ACSS2-knockdown HNSCC cells. **A** The expression levels of LC3II in shACSS2 and/or SCC9 cells overexpressing TFEB were measured by western blot and quantified statistical results. **B** The expression levels of LC3II in shACSS2 and/or CAL27 cells overexpressing TFEB were measured by western blotting and quantified statistical results. **C** The expression levels of TFEB and SQSTM1 in shACSS2 SCC9 cells overexpressing TFEB or NC were measured by western blot and quantified statistical results. **D** The expression levels of TFEB and SQSTM1 in shACSS2 CAL27 cells overexpressing TFEB or NC were measured by western blotting and quantified statistical results. **E** Fluorescence intensity of DQ-BSA staining in shACSS2 and/or SCC9 overexpressing TFEB. **F** Fluorescence intensity of LysoSensor Green staining in shACSS2 and/or SCC9 overexpressing TFEB. **G** Fluorescence intensity of DQ-BSA staining in shACSS2 and/or TFEB-overexpressed CAL27. **H** Fluorescence intensity of LysoSensor Green staining in shACSS2 and/or CAL27 overexpressing TFEB. *p < 0.05,**p < 0.01, ^#*#*^*p* < 0.01.

To clarify the role of TFEB, we overexpressed TFEB in SCC9 and CAL27 of ACSS2 knockout. TFEB overexpression also significantly improved the migration capacity of SCC9 in ACSS2 knockout, but did not seem to have a significant effect on the migration capacity of CAL27 cells (Fig. 6A, B). Transwell invasion and clone formation assays of both cell types showed that TFEB partially reversed the invasion and proliferation of HNSCC cells inhibited by ACSS2 knockdown (Fig. 6C–F). Similarly, after overexpression of TFEB, the expression of LC3II in shACSS2 HNSCC, while the expression of SQSTM1 did not show a significant increase (Fig. 7A–D), and the fluorescence intensities of DQ-BSA and LysoSensor Green were significantly enhanced, indicating that the lysosomal function and autophagic flux of head and neck squamous cell carcinoma cells were partially improved (Fig. 7E–H).

### ACSS2 KD inhibits tumor growth and autophagy in vivo

**Fig. 8:**
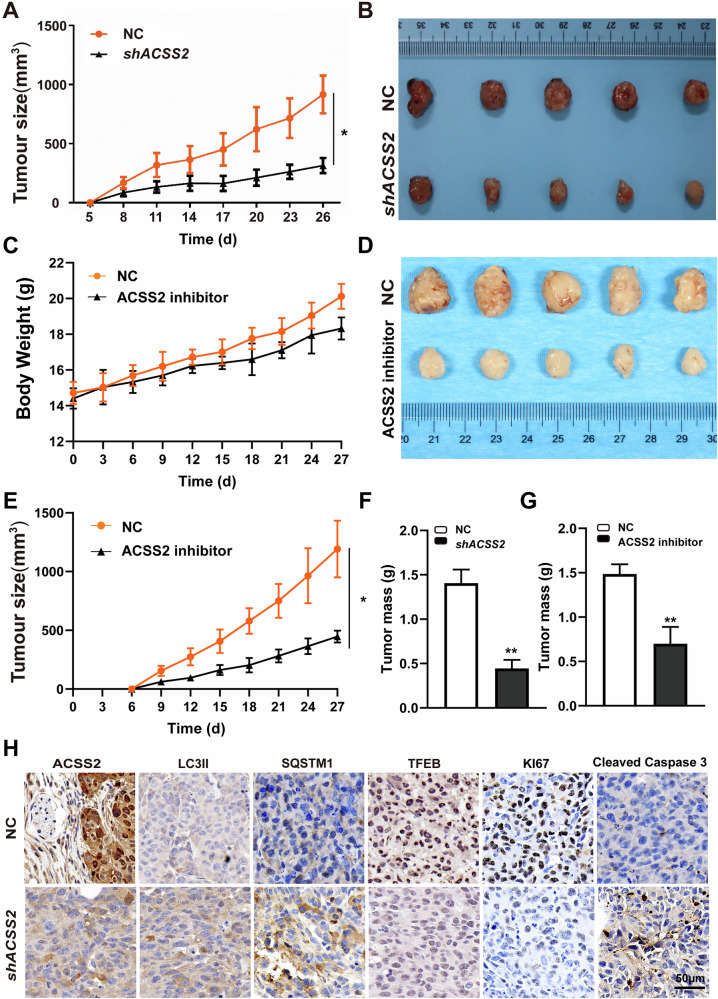
Inhibition of tumor growth and autophagy by ACSS2 KD in vivo. **A** The effect of shACSS2 on the volume changes of transplanted tumors constructed by CAL27 cells over time, as well as the images (**B**) of tumors and tumor mass (**F**) in the control group or shACSS2 group. **C** The effects of ACSS2 inhibitors on mouse body weight, (**E**) changes in transplanted tumor volume over time, images (**D**) and tumor mass (**G**) of NC or shACS2 tumors. **H** IHC representative images of ACSS2, LC3II, SQSTM1, TFEB, KI67, and Cleaved Caspase 3 in tumor tissues of mice in shACSS2 group and NC group. Scale bar=100 μm. **p < 0.01.

Subcutaneously inject shACSS2/NC CAL27 and SCC9 cells into mice, and the results showed that ACSS2 knockout had a significant inhibitory effect on the volume of transplanted tumors, indicating that ACSS2 knockout inhibited tumor growth (Fig. 8A, B, F and Fig. S4A, B, C). Furthermore, the results of HE staining indicated that compared with the tumor tissues in the ACSS2 knockdown group, the tumor tissues in the NC group exhibited higher cellular atypia (Fig. S4D). Simultaneously, in two groups of xenograft tumor mouse models established using NC CAL27 cells, we observed that the administration of ACSS2 inhibitors markedly suppressed tumor growth, while exerting no significant impact on the body weights of the mice (Fig. 8C, D, E, G).

To further elucidate the influence of ACSS2 knockdown on autophagic flux in in - vivo experiments, we employed Western Blot to detect the expression levels of autophagy-associated proteins in tumor tissues. The findings revealed a significant downregulation in the expressions of ACSS2, LAMP1, and TFEB, whereas the expressions of LC3B and SQSTM1 were significantly upregulated (Fig. S5). Furthermore, immunohistochemical analysis provided additional validation of these results. Specifically, an upregulation in the expressions of LC3 and SQSTM1 was observed, along with a downregulation in the expressions of ACSS2 and TFEB (Fig. 8H). These data suggest that ACSS2 knockdown inhibits autophagic flux in in vivo settings as well. Of note, we also discovered that following ACSS2 knockdown, there was a notable increase in the expression of Cleaved Caspase 3 and a significant decrease in the expression of KI67 (Fig. 8H). This further substantiates the notion that ACSS2 knockdown can impede tumor growth.

## Discussion and conclusions

HNSCC is a highly malignant tumor with increased chances of morbidity and mortality rates worldwide. Despite ongoing improvements in current treatments, the prognosis of patients with HNSCC remains poor. Therefore, identifying new therapeutic targets and strategies is critical [15]. In recent years, increasing attention has been paid to the role of autophagy in tumorigenesis and development, especially in HNSCC, where abnormal autophagy is closely related to the malignant progression of tumors [16]. In our study, we explored the regulatory role of ACSS2 and TFEB in the autophagy mechanism. Using molecular biology, cell biology, and clinical sample analysis, we found that ACSS2 and TFEB have a close functional axis that plays an important regulatory role in autophagy in HNSCC.

Autophagy is the mechanism by which organelles and macromolecules are degraded to recycle energy [17]. Autophagy can prevent cancer development in the initial stages; however, as the tumor progresses, it can help tumor cells survive under metabolic pressure and promote invasion and metastasis [18]. ACSS2 plays an important role in tumor autophagy, similar to that in renal cell carcinoma, where it enhances tumor proliferation and invasion by promoting LAMP1 expression [19]. As an autophagy factor, LAMP1 not only promotes cell invasion and metastasis, but also cooperates with other factors to induce lysozyme cell death and enhance drug resistance in tumor cells [20]. ACSS2 knockdown reduced the mRNA and protein levels of LAMP1 and reduced the invasion and metastasis of renal cell carcinoma [19]. Liang et al. [21]. showed that the overexpression of ACSS2 in breast cancer cells led to an increase in H3K27 acetylation levels in the ATG5 promoter region, thereby maintaining the stability of autophagy flux and counteracting the enhancement of cell proliferation, migration and invasion induced by cadmium. A similar conclusion was drawn in this study. First, we found in clinical samples that the expression level of ACSS2 in HNSCC tissues was significantly higher than that in normal tissues, and was closely related to lymph node metastasis and poor prognosis of tumors. This finding suggests that ACSS2 plays an important role in the malignant progression of HNSCC. Furthermore, through in vitro experiments, we found that knocking down the expression of ACSS2 significantly inhibited the proliferation, migration, and invasion of HNSCC cells. At the same time, we also observed that after knocking down ACSS2, the autophagy flux of cells was significantly affected, which was manifested by increased LC3II/LC3I ratio and P62 expression. These results suggest that ACSS2 influences the biological behavior of HNSCC cells by regulating autophagy.

In the autophagy process, autophagosome formation, autophagosome and lysosome fusion, and normal lysosome function are three crucial links, and abnormalities in any link may affect autophagy flux [22]. Yang et al. [23]. found that an autophagy-related cancer-suppressing peptide (ARCSP) prevented the fusion of autophagosomes and lysosomes by inhibiting the endosomal maturation of cervical cancer cells and increasing the pH of lysosomes. The resulting non-fusion autophagy volume aggregation further exacerbated toxic cell death. To determine the role of ACSS2 in autophagy, we evaluated its effect on autophagosome formation and maturation/autophagosome and lysosome fusion/lysosome functions in HNSCC cells. The results showed that knockdown of ACSS2 inhibited lysosomal function in HNSCC cells, but did not interfere with autophagosome formation or maturation and autophagosome-lysosome fusion in HNSCC cells.

To further explore the specific mechanism of ACSS2 regulation of autophagy, we studied the interaction between ACSS2 and TFEB. TFEB belongs to the microphthalmia/transcription factor E (MiT/TFE) family and is the main lysosomal transcriptional regulator [24]. TFEB can enter the nucleus and bind to specific DNA sequences, namely Coordinated Lysosomal Expression and Regulation (CLEAR) elements, thereby directly activating the expression of a series of genes related to lysosomal function and autophagy. Thus, autophagy is regulated and the fusion of autophagosomes and lysosomes is promoted [25]. We found that in HNSCC cells, when ACSS2 was knocked down, the activity of TFEB also decreased, resulting in reduced expression of autophagy-related genes, thus affecting the normal progression of autophagy. However, after TFEB overexpression, a series of autophagy-related genes were up-regulated, promoting the smooth progression of autophagic flux and partially reversing the changes in autophagic flux mediated by ACSS2KD. In addition, we confirmed the role of the ACSS2-TFEB axis in HNSCC growth using in vivo experiments. ACSS2 knockdown significantly inhibited the growth and autophagy of HNSCC cells in vivo. This finding further supports the importance of the ACSS2-TFEB axis in the malignant progression of HNSCC.

In summary, our study reveals the ACSS2-TFEB axis is a key regulator of autophagy in HNSCC. Autophagic flux is maintained by the activation of TFEB transcription, and ACSS2 supports the proliferation and invasion of HNSCC cells. Therefore, therapeutic strategies targeting the ACSS2-TFEB axis are expected to provide new treatment options and improve outcomes in patients with HNSCC. In the future, we will continue to study the molecular mechanism and clinical application of this axis to bring about new breakthroughs in the treatment of HNSCC.

## Supplementary information

aj-checklist

supplementary files

Western Blot

## Data Availability

The data supporting the findings of this study can be obtained from the corresponding author upon reasonable request.
