# Supplementary material for: ACSS2-TFEB axis acts as a critical regulator of the autophagic machinery in head and neck squamous cell carcinoma
Source: Cell Death Dis. 2025 Aug 26;16(1):650. doi: 10.1038/s41419-025-07971-9 (PMC12381122; doi:10.1038/s41419-025-07971-9)

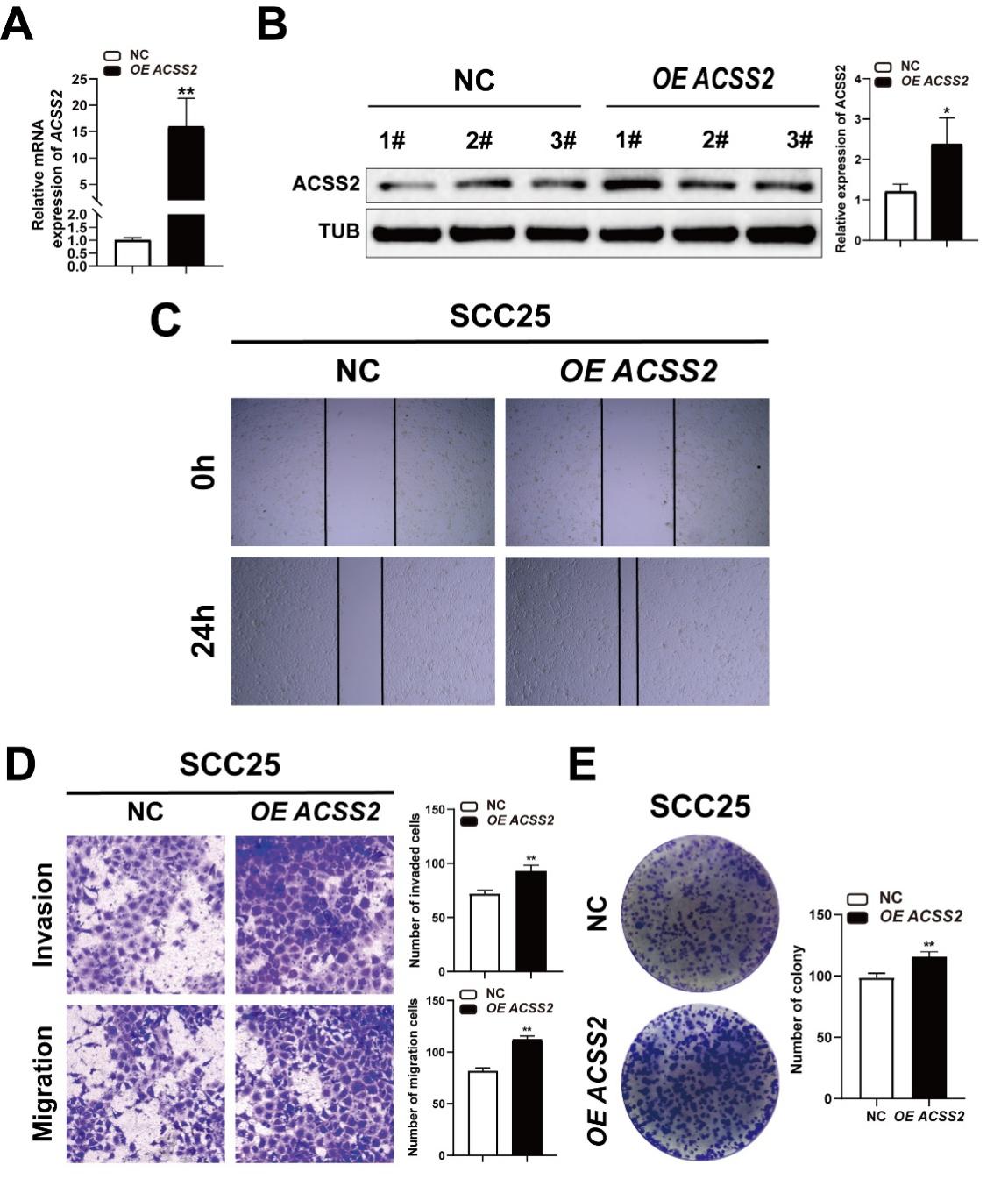


**Figure S1 The effect of ACSS2 overexpression on the proliferation, migration, and apoptosis of SCC25 cells** (A)The overexpression of ACSS2 in SCC25 cells was validated by PCR and (B)western blotting. (C) Wound healing results of ACSS2 overexpression SCC25 at 0h and 24h. (D)Transwell invasion images of ACSS2 overexpression SCC25 at 72h and quantified statistical results. Scale bar=100 μm.(E) ACSS2 overexpression SCC25 clone formation images and quantified statistical results.*p < 0.05 ,**p < 0.01.


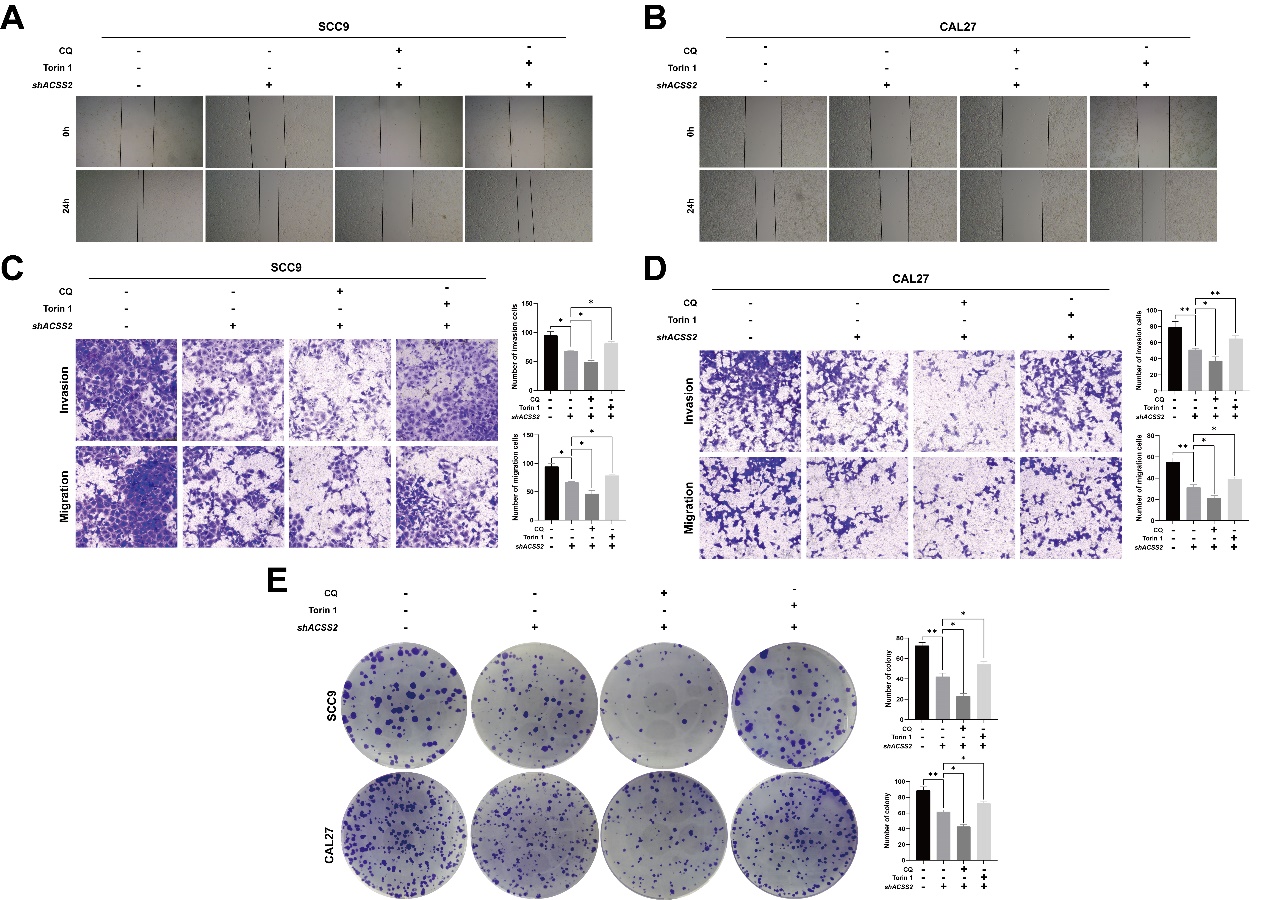
 **Figure S2 The effects of autophagy inhibitors and activators on proliferation, migration, and invasion of ACSS2 knockdown cells** (A) The wound healing results of ACSS2 knockdown/NC SCC9 treated with autophagy inhibitors and activators at 0 and 24 hours. (B) The wound healing results of ACSS2 knockdown/NC CAL27 treated with autophagy inhibitors and activators at 0 and 24 hours. (C) Transwell invasion images of ACSS2 knockdown/NC SCC9 and (D)CAL27 treated with autophagy inhibitors and activators at 72h and quantified statistical results. Scale bar=100 μm. (E) ACSS2 knockdown/NC SCC9 and CAL27 treated with autophagy inhibitors and activators clone formation images and quantified statistical results. *p < 0.05, **p < 0.01.


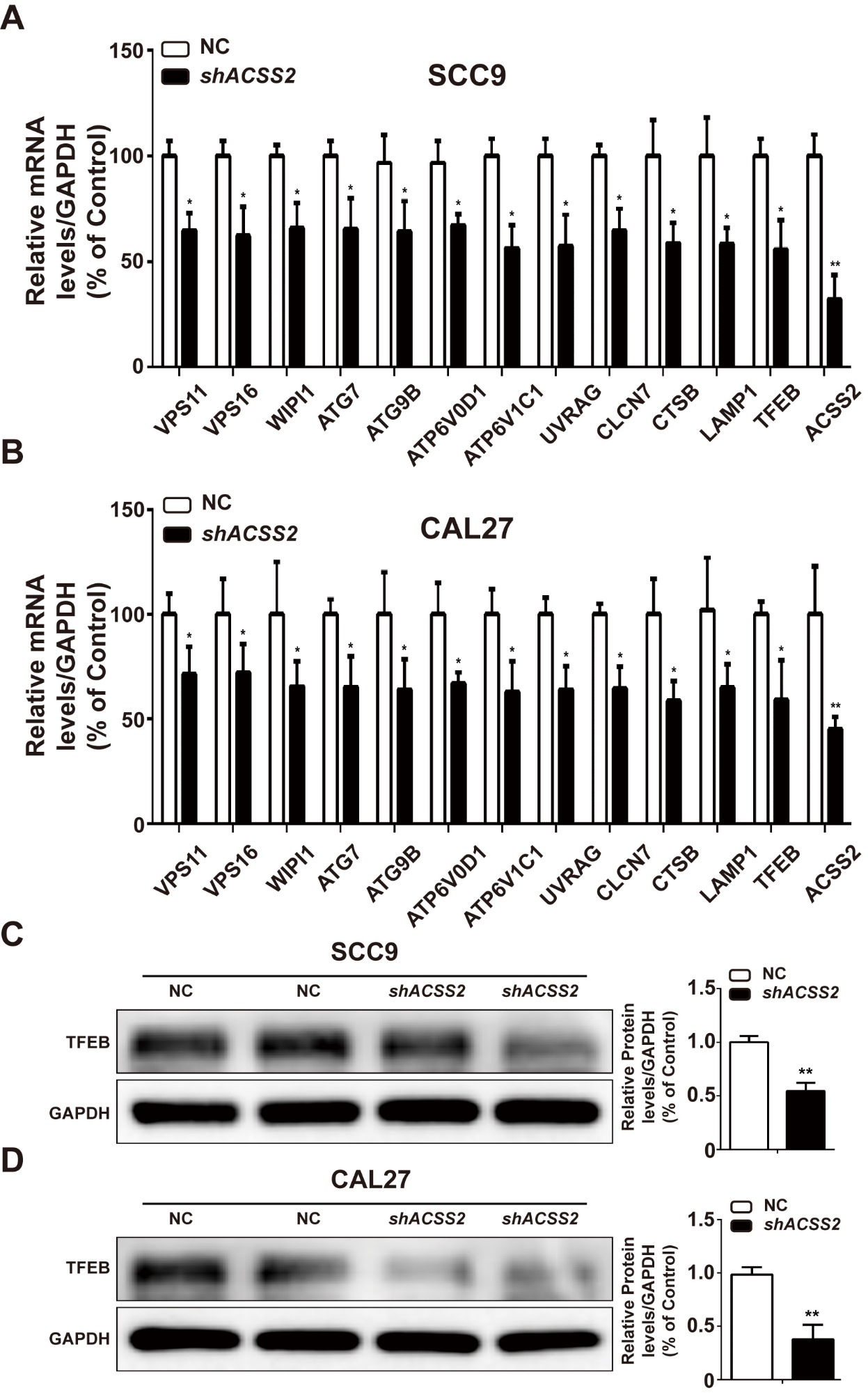
 **Figure S3 The effect of TFEB in ACSS2 knockout HNSCC cells**

1. The expression level of autophagy-related genes in SCC9 cells transfected with sh-ACSS2 was measured by qPCR and the quantitative statistical results were obtained. (B) The expression level of autophagy-related genes in sh-ACSS2-transfected CAL27 cells was measured by qPCR and the quantitative statistical results were obtained. (C) The expression level of TFEB in SCC9 cells transfected with sh-ACSS2 was measured by western blotting and quantified statistical results. (D) The expression level of TFEB in sh-ACSS2-transfected CAL27 cells was measured by western blotting and quantified statistical results. ***p* < 0.01.


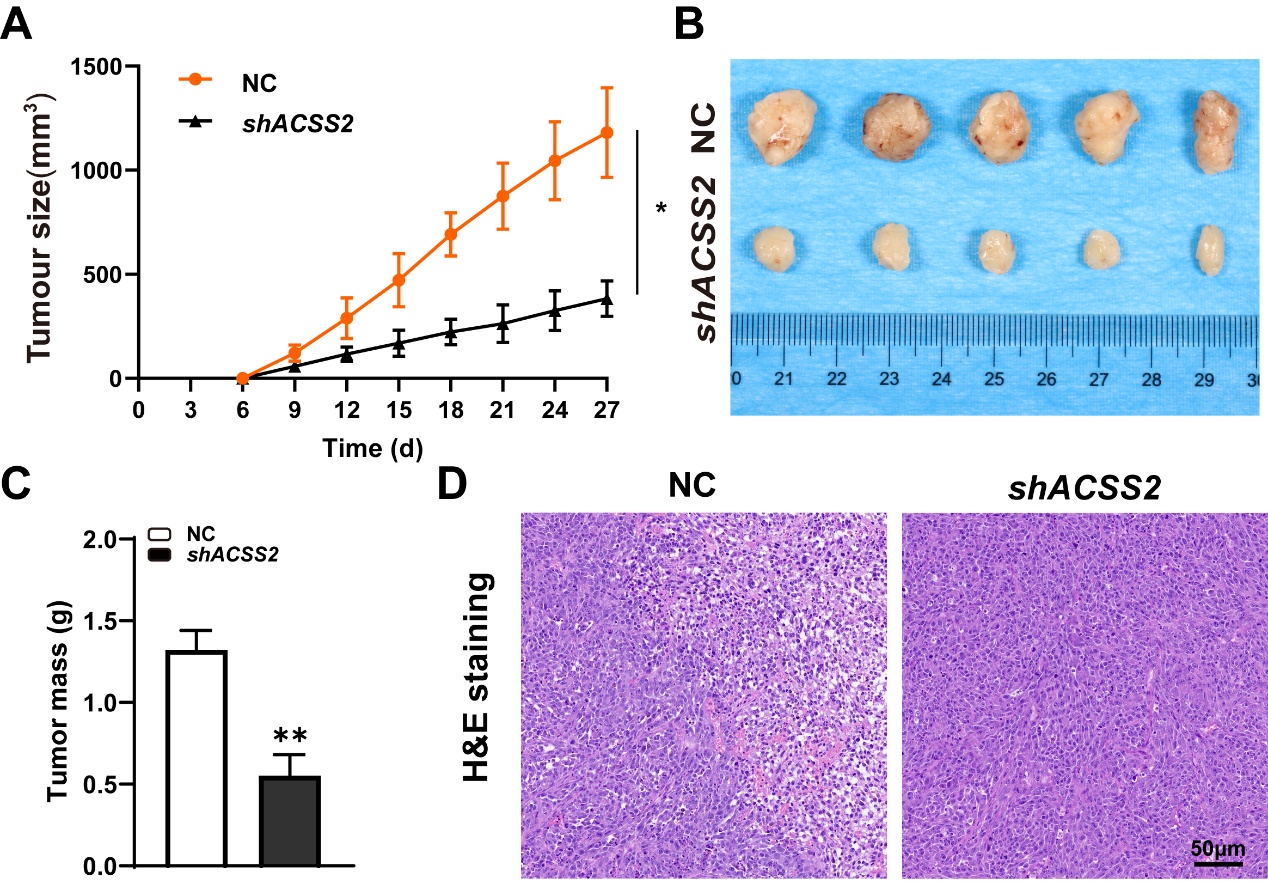


**Figure S4 Inhibition of tumor growth by shACSS2 in vivo**

(A) The effect of shACSS2 on the volume changes of transplanted tumors constructed by SCC9 cells over time, as well as the images (B) of tumors and tumor mass (C) in the control group or shACSS2 group. (D)H&E representative images in tumor tissues of mice in shACSS2 group and control group. Scale bar=50μm. *p < 0.05, **p < 0.01.


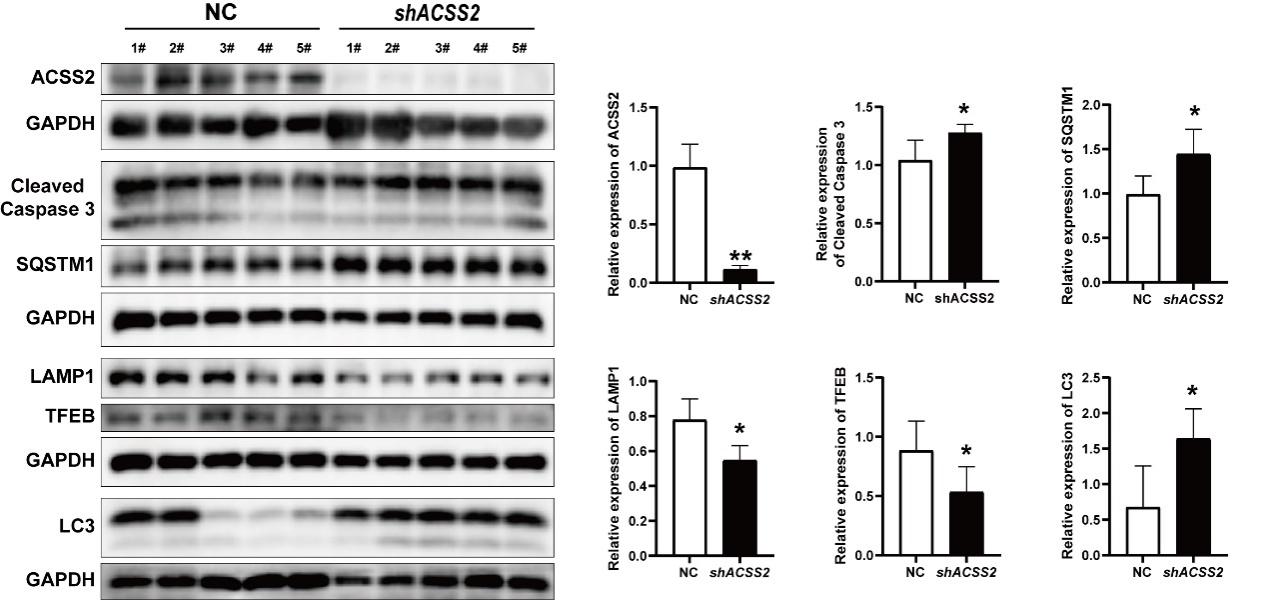
 **Figure S5 Inhibition of tumor autophagy by shACSS2 in vivo**

The expression levels of ACSS2, Cleaved Caspase 3, SQSTM1, LAMP, TFEB, and LC3II in tumor tissues of ACSS2 knockout mice using Western blotting, and the quantitative statistical results were obtained.


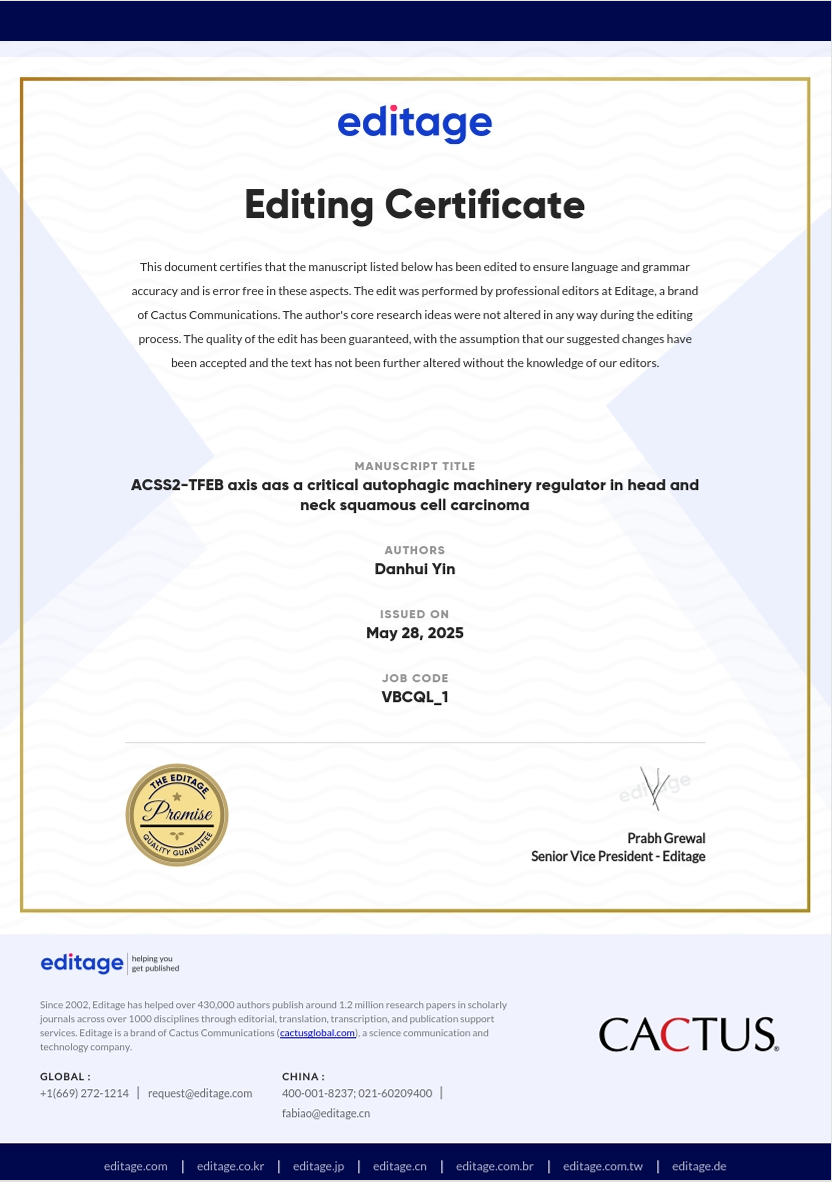

Supplement: Supplementary file 2 — supplementary files [file 41419_2025_7971_MOESM2_ESM.docx]
