# Supplementary figures and images for: ACSS2-TFEB axis acts as a critical regulator of the autophagic machinery in head and neck squamous cell carcinoma
Source: Cell Death Dis. 2025 Aug 26;16(1):650. doi: 10.1038/s41419-025-07971-9 (PMC12381122; doi:10.1038/s41419-025-07971-9)

**All full western blot data in the manuscript**


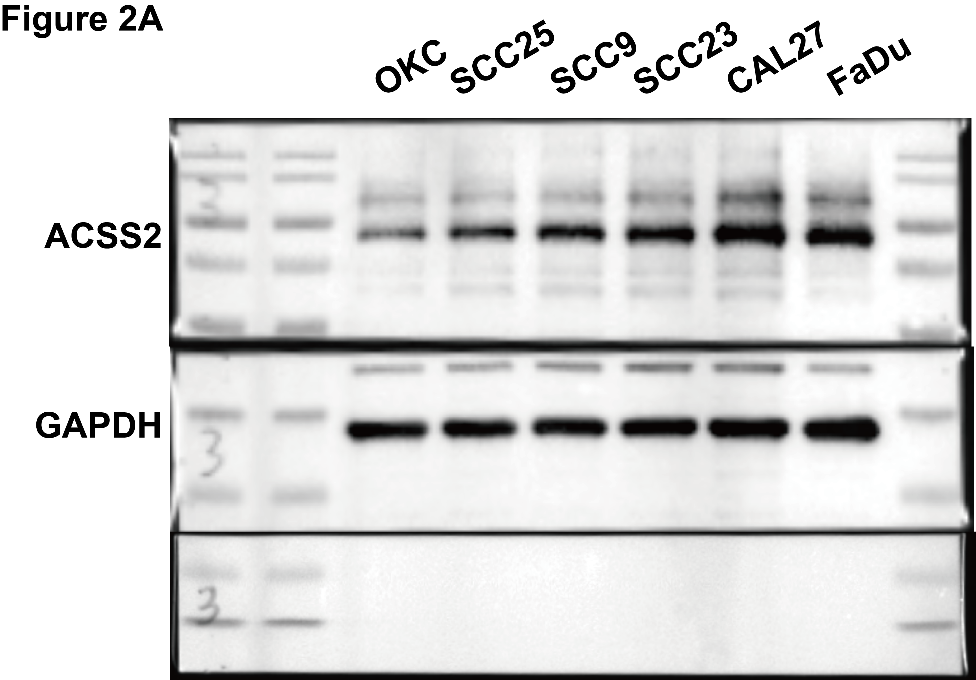


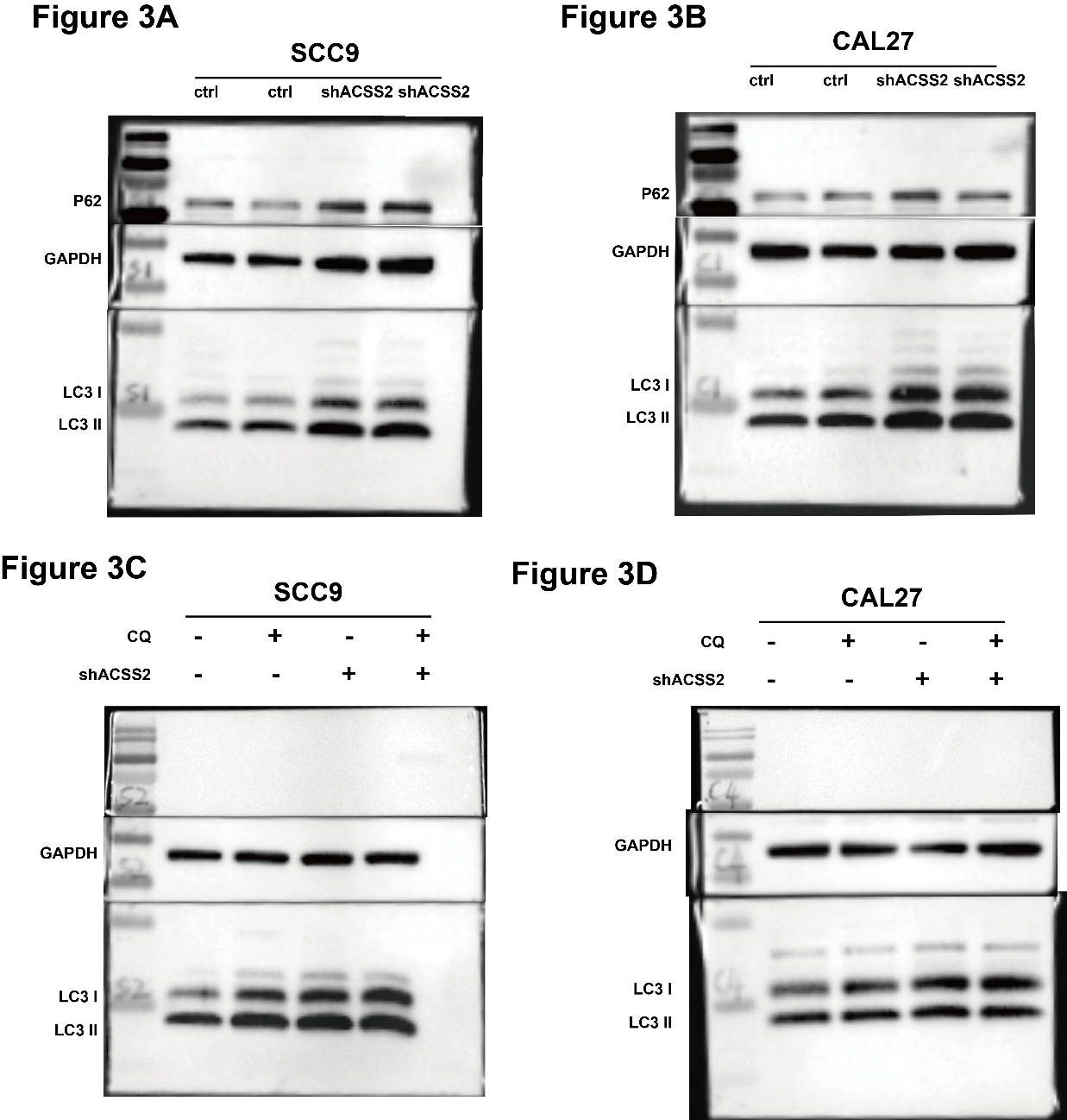


**
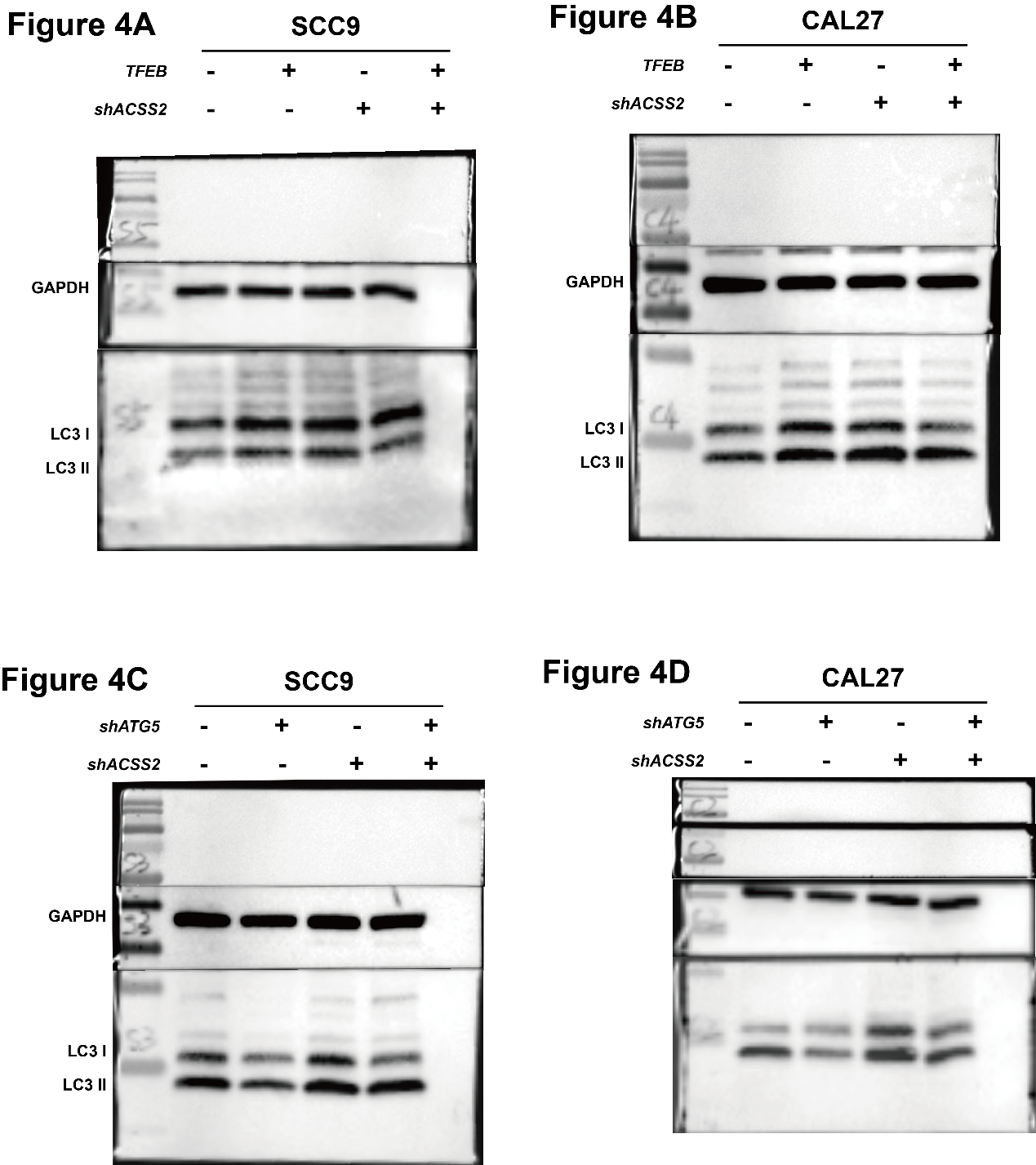
**
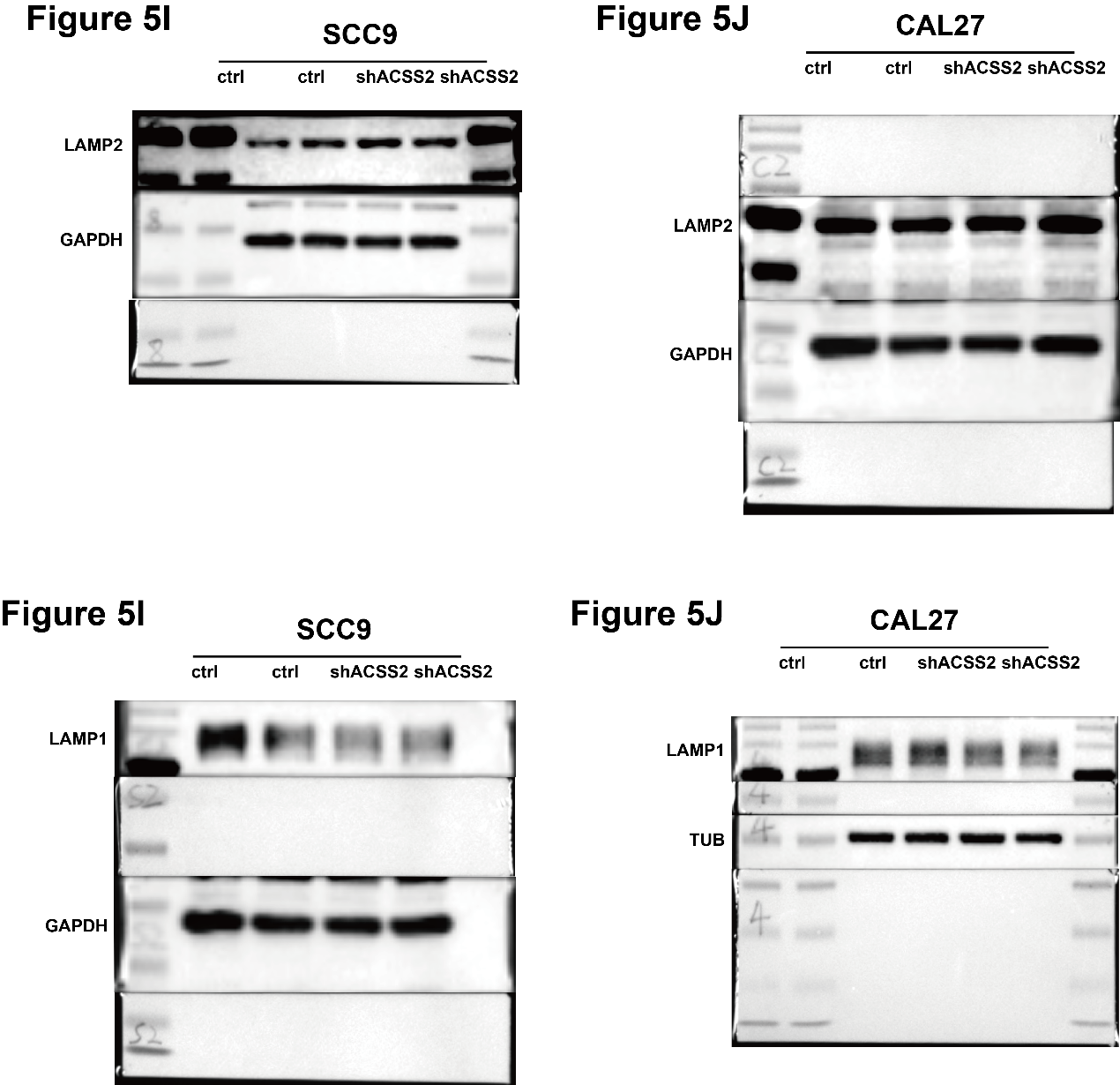

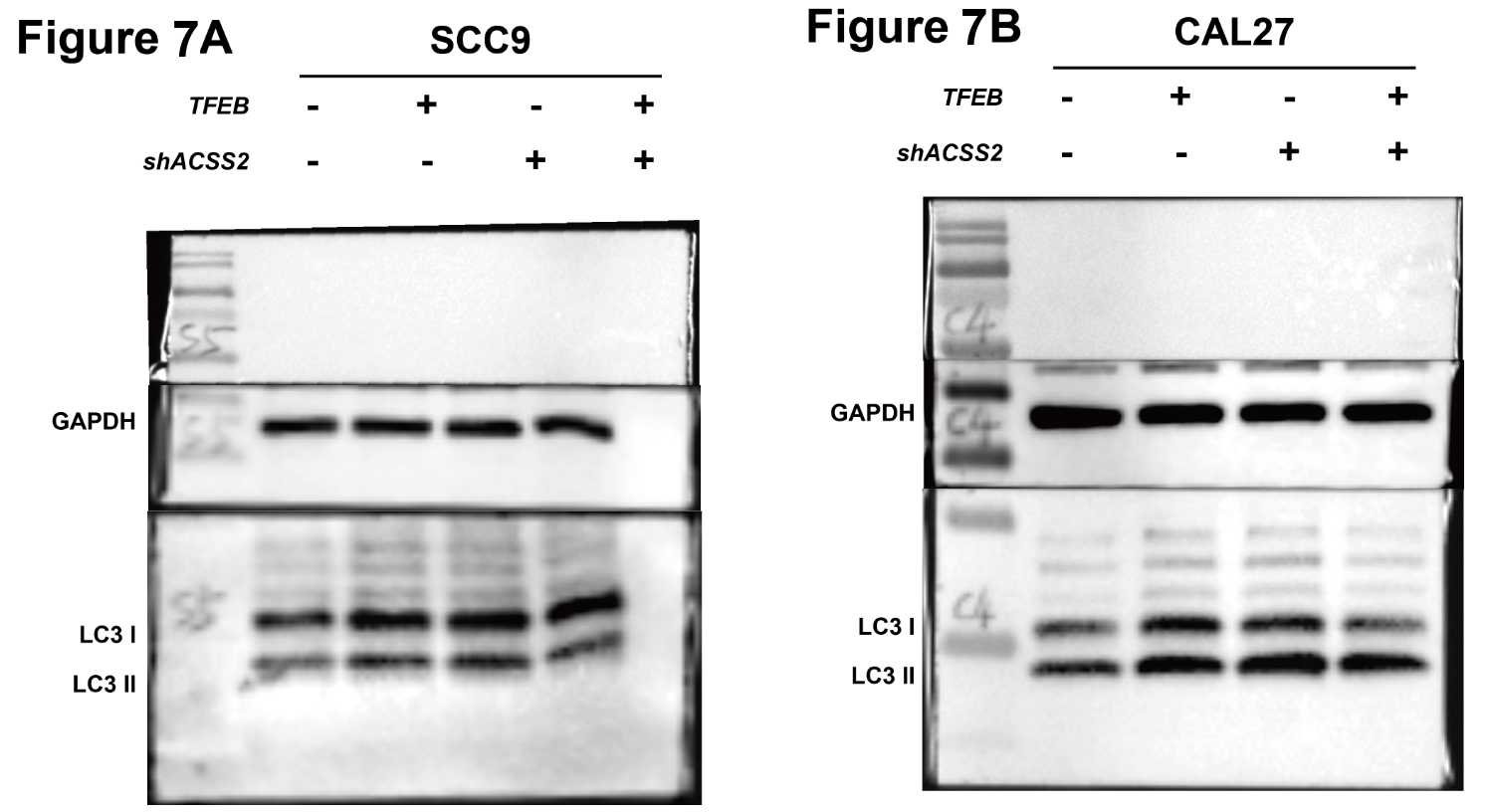

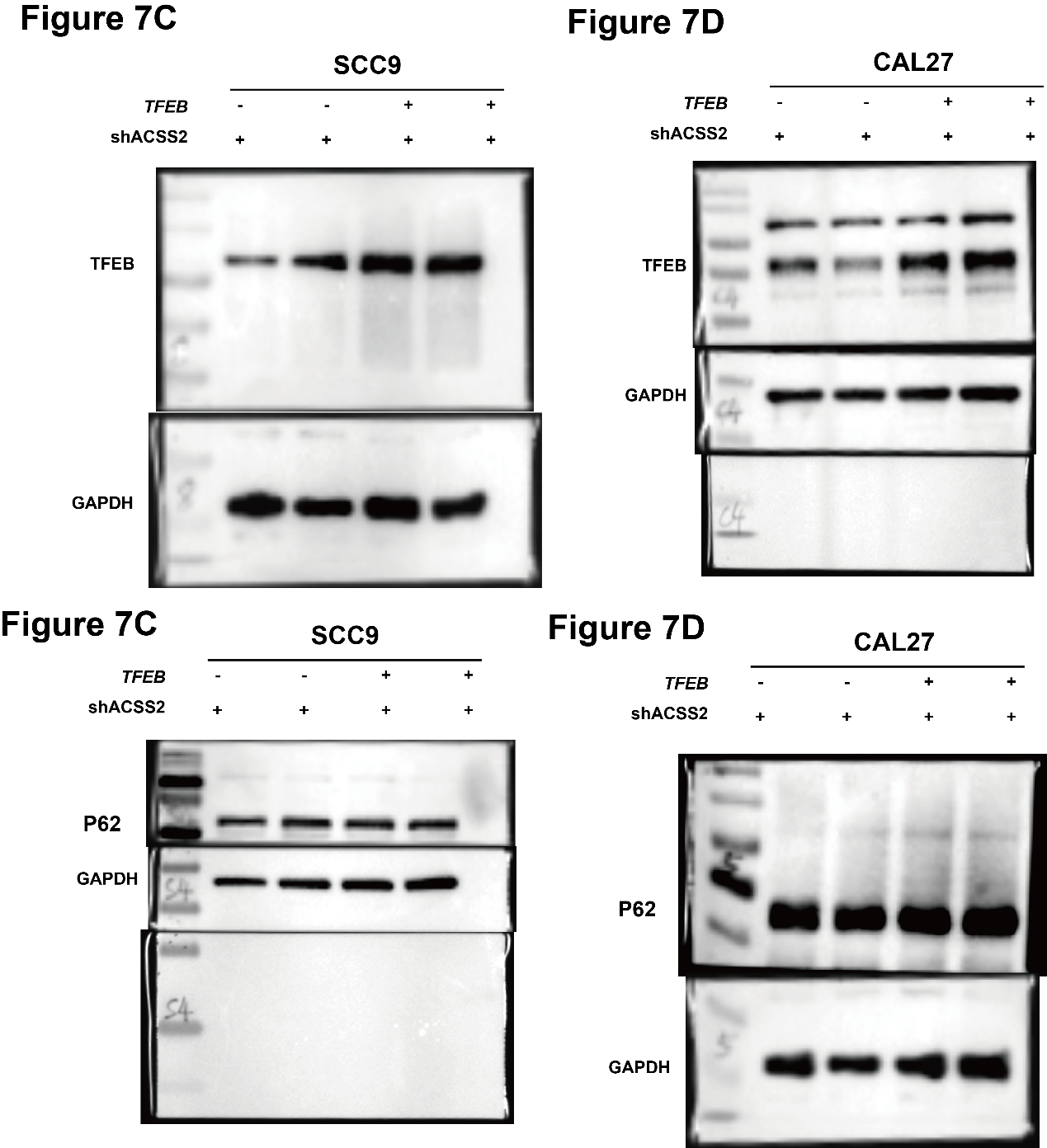


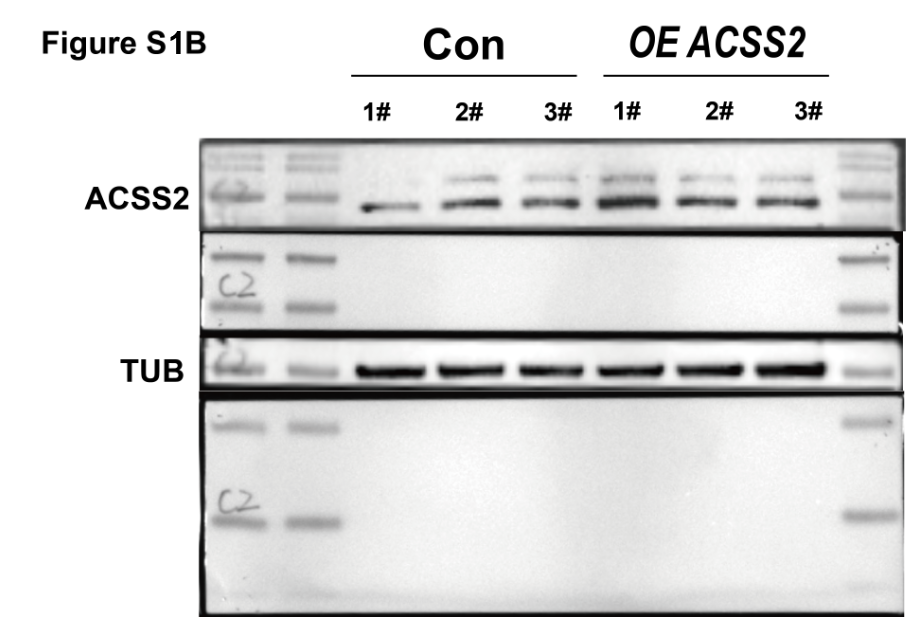


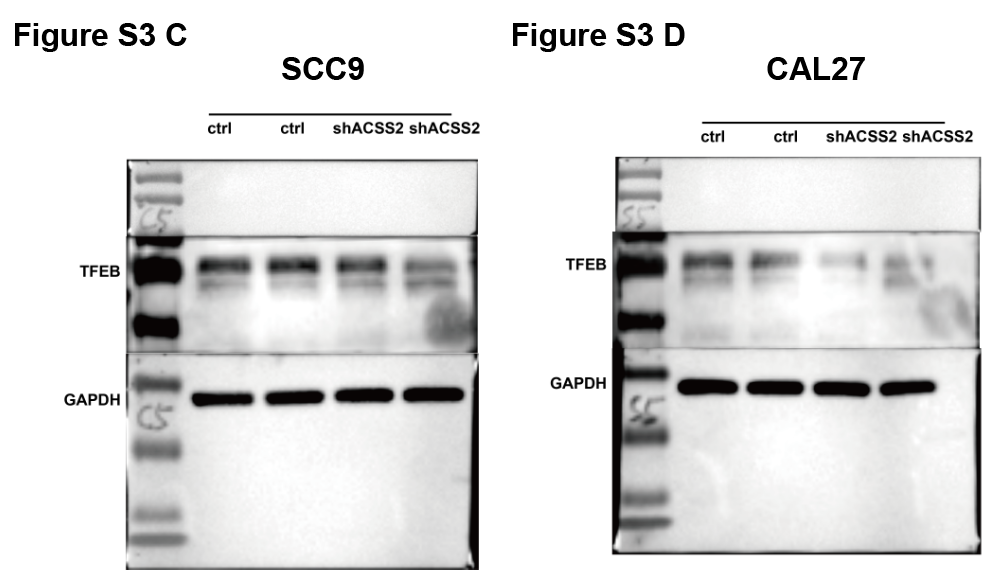


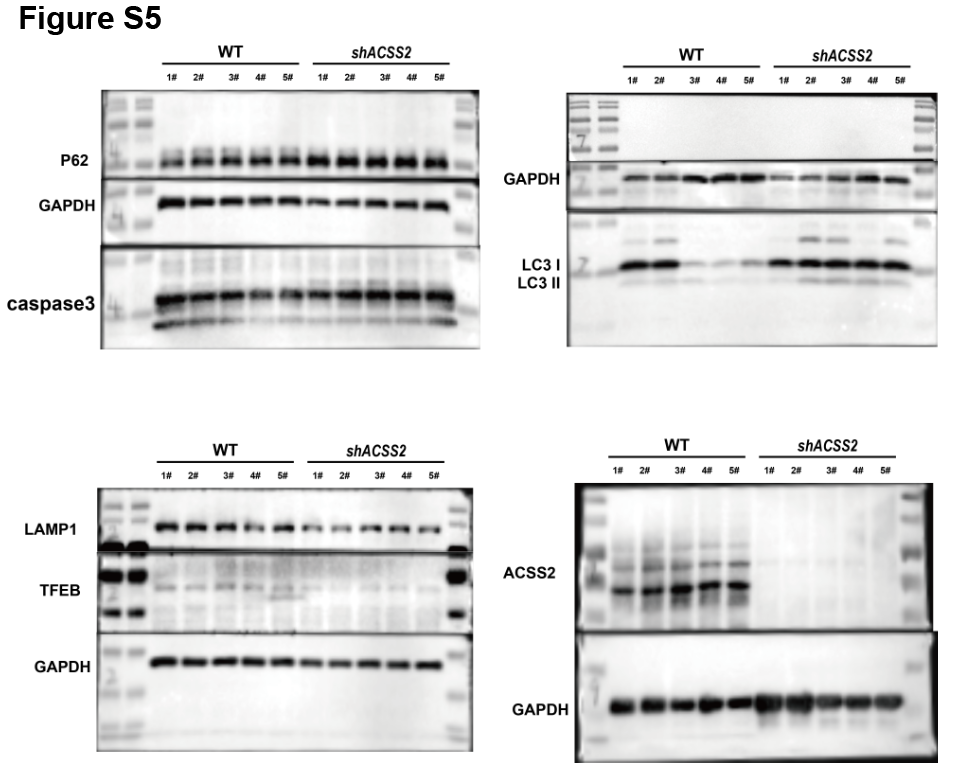

Supplement: Supplementary file 3 — Western Blot [file 41419_2025_7971_MOESM3_ESM.docx]
